# Vasorin (VASN) overexpression promotes pulmonary metastasis and resistance to adjuvant chemotherapy in patients with locally advanced rectal cancer

**DOI:** 10.1186/s12967-024-05473-4

**Published:** 2024-08-06

**Authors:** Da Kang, Shanshan Huang, Yijun Liao, Siyuan Mi, Jingying Zhou, Yu Feng, Riming Huang, Zhen-hai Lu, Z. Z. Pan, Wenjuan Ma, Gong Chen, Jia-Xing Yue, Jingxiu Huang, R. X. Zhang

**Affiliations:** 1 Department of Colorectal Surgery, State Key Laboratory of Oncology in South China, Sun Yat-sen University Cancer Centre, Guangzhou, Guangdong 510060 P. R. China; 2 State Key Laboratory of Oncology in South China, Guangdong Key Laboratory of Nasopharyngeal Carcinoma Diagnosis and Therapy, Guangdong Provincial Clinical Research Center for Cancer, Sun Yat-sen University Cancer Center, Guangzhou, Guangdong 510060 China; 3 Department of Anesthesiology, Sun Yat-sen University Cancer Centre, Guangzhou, Guangdong 510060 China; 4 Department of Breast Oncology, Sun Yat-sen University Cancer Center, Guangzhou, 510060 Guangdong Province China; 5 School of Biomedical Sciences, Chinese University of Hong Kong, Hong Kong SAR, 999077 China; 6 BGI-Shenzhen, Shenzhen, 519103 P. R. China; 7 Guangdong Provincial Key Laboratory of Food Quality and Safety, College of Food Science, South China Agricultural University, Guangzhou, 510642 China; 8 Department of Intensive Care Unit, Sun Yat-sen University Cancer Centre, Guangzhou, 510060 Guangdong China; 9 Department of Anesthesiology, State Key Laboratory of Oncology in Southern China, Collaborative Innovation Center for Cancer Medicine, Sun Yat-sen University Cancer Centre, Guangzhou, 510060 Guangdong P. R. China

**Keywords:** Rectal cancer, Pulmonary metastasis, MAPK pathway, NOTCH pathway, Adjuvant therapy

## Abstract

**Background:**

LARC patients commonly receive adjuvant therapy, however, hidden micrometastases still limit the improvement of OS. This study aims to investigate the impact of VASN in rectal cancer with pulmonary metastasis and understand the underlying molecular mechanisms to guide adjuvant chemotherapy selection.

**Methods:**

Sequencing data from rectal cancer patients with pulmonary metastasis from Sun Yat-sen University Cancer Center (SYSUCC) and publicly available data were meticulously analyzed. The functional role of VASN in pulmonary metastasis was validated in vivo and in vitro. Coimmunoprecipitation (co-IP), immunofluorescence, and rescue experiments were conducted to unravel potential molecular mechanisms of VASN. Moreover, VASN expression levels in tumor samples were examined and analyzed for their correlations with pulmonary metastasis status, tumor stage, adjuvant chemotherapy benefit, and survival outcome.

**Results:**

Our study revealed a significant association between high VASN expression and pulmonary metastasis in LARC patients. Experiments in vitro and in vivo demonstrated that VASN could promote the cell proliferation, metastasis, and drug resistance of colorectal cancer. Mechanistically, VASN interacts with the NOTCH1 protein, leading to concurrent activation of the NOTCH and MAPK pathways. Clinically, pulmonary metastasis and advanced tumor stage were observed in 90% of VASN-positive patients and 53.5% of VASN-high patients, respectively, and VASN-high patients had a lower five-year survival rate than VASN-low patients (26.7% vs. 83.7%). Moreover, the Cox analysis and OS analysis indicated that VASN was an independent prognostic factor for OS (HR = 7.4, P value < 0.001) and a predictor of adjuvant therapy efficacy in rectal cancer.

**Conclusions:**

Our study highlights the role of VASN in decreasing drug sensitivity and activating the NOTCH and MAPK pathways, which leads to tumorigenesis and pulmonary metastasis. Both experimental and clinical data support that rectal cancer patients with VASN overexpression detected in biopsies have a higher risk of pulmonary metastasis and adjuvant chemotherapy resistance.

**Supplementary Information:**

The online version contains supplementary material available at 10.1186/s12967-024-05473-4.

## Background

Rectal cancer is one of most prevalent cancers worldwide and is the leading cause of cancer-related mortality [1]. Patients with locally advanced rectal cancer (LARC) face numerous challenges, including a high risk of local recurrence, limited sphincter preservation, low long-term survival rates, and high distant metastasis rates. Previous clinical trials, including QUASAR [2], MOSAIC [3], XELOXA [4], and IDEA [5], have demonstrated that adjuvant therapy can improve overall survival (OS) in colorectal cancer (CRC) patients. However, limited data are available for patients with rectal cancer across numerous clinical cohorts [6–9]. In contrast to colon cancer, in which treatment failure is predominantly indicated by distant metastasis, first failure in rectal cancer patients can be indicated by local recurrence (pelvis) or distant metastasis (lung) [2].

For LARC patients, especially those with a high recurrence risk, neoadjuvant chemoradiotherapy is recommended. Based on the results of previous clinical trials [3–5], it is still controversial whether adjuvant chemotherapy is necessary for patients after neoadjuvant therapy. According to guidelines from the National Comprehensive Cancer Network (NCCN), postoperative chemotherapy is recommended for all patients who undergo preoperative chemoradiotherapy, even if they achieved a pathologic complete response to neoadjuvant therapy [10].

The principle of adjuvant therapy is to eliminate micrometastases at the time of surgery [11], and to best apply this approach, scientists need to a clear understanding of the molecular characteristics of tumors during the process of tumor metastasis. Metastasis is a crucial factor in determining the long-term survival of rectal cancer patients and accounts for 90% of tumor-related deaths [10, 12]. While liver metastases are more common in patients with colon cancer, pulmonary metastases are more prevalent in patients with mid-low rectal cancer [13]. Recent studies have also reported a greater incidence of pulmonary metastasis in rectal cancer patients than in colon cancer patients [14, 15].

More evidence is needed to identify which groups of patients need adjuvant chemotherapy [16]. In recent years, emerging methods for identifying high-risk patients, such as tissue biomarker profiling [17], artificial intelligence-driven digital image models [18], and circulating tumor DNA (ctDNA) measurement via liquid biopsy [19], have provided new molecular perspectives for treatment selection. Ongoing research and development focused on new approaches and treatments offer hope for continued progress in this area.

Vasorin (VASN) is a type I membrane protein that is primarily studied in blood vessels, where it contributes to vascular morphogenesis and injury response [20–22]. It can be cleaved by ADAM family metalloproteases, and its soluble form is involved in various biological functions, including inhibiting TGFβ signaling, regulating the EMT process, and inducing angiogenesis [23]. VASN has been identified as an oncogene in various cancers, such as glioma [24], thyroid cancer [25], and prostate cancer [26]. However, its role in rectal cancer metastasis, especially in lung metastasis, has not been explored, and our study reports this phenomenon and the potential underlying mechanism for the first time. Clarification of the relationships among VASN expression, pulmonary metastasis and adjuvant chemotherapy response in rectal cancer patients could provide valuable insights into the molecular mechanisms driving metastasis and might help identify the patient population most likely to benefit from adjuvant therapy.

## Methods

### Transcriptome sequencing

Between January 1st, 2008, and January 1st, 2021, we collected a total of 8 pairs of rectal cancer tissues from patients with and without pulmonary metastasis from the Sun Yat-sen University Cancer Center (SYSUCC), for second-generation transcriptome sequencing. Tumor pathological type was determined based on the classification system provided by the World Health Organization (WHO), while the tumor pathological staging followed the classification system of the American Joint Committee on Cancer (AJCC). Additionally, we collected 7 pairs of rectal cancer tissues and normal rectal epithelial tissues from rectal cancer patients during surgical resection. The study was approved by the Institutional Ethical Review Boards of SYSUCC, with reference number B2023-554-01.

### Cell culture

The human CRC cell lines (SW837, T84 and LOVO) and HEK293T cells were purchased from the American Type Culture Collection (ATCC), and all the cells were authenticated. SW837 and T84 cells were cultured in DMEM (Invitrogen) supplemented with 15% fetal bovine serum (Gibco), and HEK293T cells were cultured in DMEM (Invitrogen) supplemented with 8% fetal bovine serum (Gibco). In addition, LOVO cells were cultured in RPMI-1640 (Invitrogen) supplemented with 10% fetal bovine serum (Gibco). Penicillin‒streptomycin (Gibco, Cat# 15,140,122) was added to DMEM and RPMI-1640 (100 U/mL).

### Plasmids and transfection

The VASN coding sequence with an added HA tag was amplified and cloned and inserted into the pSin-EF2-puro empty plasmid to generate the pSin-EF2-puro-VASN-HA overexpression plasmid. The p3xFlag-CMV-NOTCH1 plasmid (human) was purchased from G-CLONE (China). The shRNA targeting VASN and the PLVX-IRES-GFP-puro vector were purchased from RiboBio (China). We performed lentiviral infection based on the manufacturer’s protocols for 48 h and then screened the cells with puromycin (1:1000) for 2 weeks to obtain VASN-knockdown cells. The shRNA sequences used were as follows:

ShVASN#1: CCGGAGCTTGACTACGCCGACTTTGCTCGAGCAAAGTCGGCGTAGTCAAGCTTTTTTG.

ShVASN#2: CCGGAGCCAACAGGCTGCATGAAATCTCGAGATTTCATGCAGCCTGTTGGCTTTTTTG.

Human CRC cell lines (SW837, T84 and LOVO) were transfected using Lipofectamine 3000 (Invitrogen, Cat# L300015), and HEK293T cells were transfected with PEI transfection reagent (MCE, Cat# HY-K2014). The transfection efficiency was assessed using RT‒qPCR after 24 h or Western blotting after 48 h.

### Real-time quantitative PCR analysis (qRT‒PCR)

For qRT‒PCR, total RNA was extracted with TRIzol reagent (Thermo Fisher, Cat# 15,596,018). HiScript III RT SuperMix for qPCR (+ gDNA wiper) (Vazyme, Cat# R323-01) was used to perform reverse transcription to obtain complementary DNA, and qPCR was performed using qPCR SYBR Green Master Mix (Yeasen, Cat# 11201ES08) on a LightCycler 480 System (Roche). The 2^−ΔΔCT^ method was used for normalization to obtain the relative gene expression. The sequences of primers used in this article were as follows:

ACTB forward: 5’-CATGTACGTTGCTATCCAGGC-3’.

ACTB reverse: 5’-CTCCTTAATGTCACGCACGAT-3’.

VASN forward: 5’-TCTCACCTATCGCAACCTATCG-3’.

VASN reverse: 5’-CAGACGGAGTAAGTGGCGTT-3’.

### Cell viability and colony formation assays

For the cell viability assay, 1000 cells per well were seeded into 96-well plates. On the indicated days, 10 µl of Cell Counting Kit-8 (CCK-8) reagent (GLPBIO, Cat#GK10001) was added to each well, after which the absorbance at 450 nm was measured via a spectrophotometer after incubation at 37 °C for 2 h. For the drug sensitivity assay, cells in 96-well plates were cultured in media supplemented with different concentrations of 5-fluorouracil (5-FU) for 48 h. Then, a cell viability assay was performed, and the half-maximal inhibitory concentration (IC_50_) was subsequently calculated. For the colony formation assay, 800 cells per well were plated into 6-well plates. After stable colony formation (~ 14–20 days), the colonies in the 6-well plates were fixed with methanol for 30 min–1 h and then stained with crystal violet for 2 h. The counts of colonies that contained more than 50 cells were measured using ImageJ software.

### Migration and invasion assays

For the migration assay, 5 × 10^4^ cells were suspended in 200 µl serum-free medium and then inoculated into transwell chambers (8-µm pores; Corning, Cat# 354,480). The chambers were placed into 24-well plates containing 500 µl of complete medium supplemented with 16% FBS and cultured at 37 °C for 8–12 h. For the invasion assay, 1 × 10^5^ cells were plated into chambers precoated with Matrigel (BioCoat, Cat# 354,262) (invasion assay) and cultured at 37 °C for 20–24 h. The cells in the chambers were fixed using methanol for 30 min–1 h and then stained with crystal violet for 2 h. Random visual fields were obtained using a microscope (Lecia) and further analyzed using ImageJ.

### Wound healing assay

Cells in 6-well plates were cultured in complete medium until they reached 90% confluence, after which 10 µl sterile tips were used to scratch a line across the cell monolayer. The suspended cells were removed by washing the 6-well plates three times with PBS, and the attached cells were subsequently cultured in FBS-free medium for 24–72 h. At the indicated times, images of the wounded areas were obtained using a microscope (Lecia) and further analyzed using ImageJ.

### Western blotting

The cells were lysed on ice for 15 min using 1x RIPA lysis buffer (Merck Millipore, Cat#20–188) supplemented with 50x protease inhibitors and 50x phosphatase inhibitors (Beyotime, Cat#P1046). The lysates were then centrifuged to obtain the total protein concentration. For fresh-frozen specimens, the tissues were ground in liquid nitrogen and then lysed to obtain total protein. The proteins were added to 5x loading buffer (FDbio, Cat# FD006) and boiled at 100 °C for 10 min. Proteins were separated through SDS‒PAGE (EpiZyme) and then transferred to 0.22–0.45 μm PVDF membranes (Merck Millipore). The membranes were blocked with 5% nonfat milk for 1 h and incubated with the following primary antibodies: anti-β-actin (Proteintech, Cat# 20536-1-AP, 1:1000), anti-VASN (Abcam, Cat# ab156868, 1:1000), anti-N-cadherin (MCE, Cat# HY-P80238, 1:1000), anti-E-cadherin (MCE, Cat# HY-P80112, 1:1000), anti-β-catenin (MCE, Cat# HY-P80488, 1:1000), anti-CDK1 (Affinity Biosciences, Cat# CDK1/CDC2, 1:1000), anti- Cyclin B1 (Affinity Biosciences, Cat# Cyclin B1, 1:1000), anti-NOTCH1 (CST, Cat# 3608 S, 1:1000), anti-NICD (NOTCH1 intracellular domain, cleaved form of NOTCH1) (CST, Cat# 4147T, 1:1000), anti-MAPK (CST, Cat# 9926T, 1:1000), and anti-phospho-MAPK (CST, Cat# 9910T, 1:1000) antibodies overnight. Next, the membranes were incubated with HRP-conjugated secondary antibodies (Proteintech, Cat# SA00001-1/SA00001-2, 1:5000) for 1 h, and then the protein expression levels were visualized via chemiluminescence.

### Coimmunoprecipitation (co-IP) assays

HEK293T and CRC cell lines were used for protein interaction-related experiments. The cells were lysed on ice for 30 min using IP lysis buffer (Beyotime, Cat# P0013), and the lysates were immunoprecipitated with 3 µg of the indicated antibodies (anti-HA, Sigma, Cat# H6908; anti-Flag, Sigma, Cat# F1804) at 4 °C overnight. The immune complex was captured by Protein A/G Magnetic beads (MCE, Cat# HY-K0202A) and then washed with IP wash buffer. The protein-bead complexes were added to 1x loading buffer and boiled at 100 °C for 10 min. The eluates were separated by SDS‒PAGE, and the proteins were detected via Western blot analysis.

### Flow Cytometry

The staining of cell cycle and apoptosis by flow cytometry was performed using commercially available kits (GOONIE, Cat# 100–107, Elabscience, Cat# E-CK-A211). SW837, LOVO and T84 cells were collected and incubated with PI for cell cycle and FITC-Annexin-V/PI solution for apoptosis. Cells were analyzed using flow cytometry (CytoFLEX, Beckman Coulter) and the data were analyzed with FlowJo software.

### Immunofluorescence staining

Cells were seeded into 24-well plates precoated with glass slides until the cell density reached 50-70%, followed by fixation with 0.4% paraformaldehyde for 1 h and permeabilization with 0.5% Triton X-100 for 20 min. The cells were then blocked for 1 h with QuickBlock™ Blocking Buffer for Immunofluorescence (Beyotime, Cat# P0260) and incubated with anti-rabbit VASN (CUSABIO, Cat# CSB-PA025796OA01HU, 1:100) and anti-mouse NOTCH1 (Abmart, Cat# P90415R1S, 1:100) at 4 °C overnight, followed by incubation with secondary antibodies (Alexa Fluor 488 and Alexa Fluor 549) at RT for 1 h and staining with Honest for 3 min. The images were obtained using a confocal laser scanning microscope and analyzed using ZEN 3.1 (blue edition).

### In vivo tumor model

Female BALB/c nude mice (6 weeks old) were purchased from Guangdong GemPharmatech. To establish the subcutaneous tumorigenesis model, the mice were subcutaneously injected with LOVO/SW837 cells stably overexpressing 1 × 10^6^ VASN and the corresponding control cells. The tumor volume (0.5 × length × width^2^) was measured and recorded every three days beginning on day 7. Mice were sacrificed if the maximum diameter of the tumors in any group was greater than 1.5 cm, and the tumor tissue was paraffin-embedded for hematoxylin and eosin (H&E) staining and immunohistochemistry (IHC) after the tumors were weighed. A pulmonary metastasis model was established by intravenous injection of luciferase-labeled LOVO/SW837 cells. After 2 weeks, pulmonary metastases were observed with the Living Image software (PerkinElmer). The protocols for all the animal experiments in this study were approved by the Experimental Animal Ethics Committee of Sun Yat-sen University Cancer Center (L025504202212007).

### Hematoxylin and eosin (H&E) staining and immunohistochemistry (IHC)

The paraffin sections were deparaffinized at 65 °C for 1 h and then rehydrated (100% alcohol for 1 min-95% alcohol for 1 min-80% alcohol for 1 min-60% alcohol for 1 min-ddH_2_O for 1 min). Endogenous peroxidase activity was blocked using 3% H2O2 for 15 min, after which the tissue antigen was removed with high-temperature citrate. For H&E staining, the sections were immersed in hematoxylin and eosin. For IHC, the sections were incubated with primary antibodies (anti-VASN, Antibody Systems, Cat#PHJ05101, 1:100; anti-phospho-ERK, CST, Cat#9910T, 1:100) at 4 °C overnight and with HRP-conjugated rabbit/mouse secondary antibodies (BOSTER, Cat#SA1020-1/2 Kit) at room temperature for 1 h. The sections were finally stained with DAB (BOSTER, Cat# SA1020-1/2 Kit) and counterstained with hematoxylin. Two experienced pathologists analyzed the sections. The staining intensity was scored as follows: negative staining-0, weak staining-1, moderate staining-2, and strong staining-3. The staining intensity was classified according to the percentage of positive cells: <10%-1, 10 ~ 35%-2, 35 ~ 70%-3, and > 70%-4. The staining intensity and the staining degree were multiplied to calculate the IRS.

### Bioinformatics analysis

The RNA-seq data were subjected to comprehensive analysis using an in-house pipeline to quantify, normalize, and compare transcripts. In summary, human reference genome assemblies and corresponding annotations were downloaded from ENSEMBL and GENCODE. Subsequently, gffread v0.12.2 and Salmon were used to extract and quantify the human reference transcriptome. Finally, downstream analysis of the quantified counts was conducted using the DESeq2 and WGCNA packages in 4.2.2 R version, and data visualization was based on the ggplot2, GseaVis and corrplot packages.

### Statistical analysis

The data in this study are presented as the mean ± S.D and data from experiments were replicated three times or more for statistical analysis. All the statistical analyses in this study were performed using GraphPad Prism 9. The P value for two independent groups was calculated using two-tailed unpaired Student’s test or Wilcoxon rank sum test. The P value for multiple comparisons was calculated using one-way or two-way ANOVA. The normality and frequency tests were conducted before the Student’s t-test and ANOVA. Survival analysis was performed using the Kaplan‒Meier method and log-rank analysis. The P value < 0.05 indicated statistical significance.

## Results

### The relationship between VASN expression and pulmonary metastasis in rectal cancer patients

**Fig. 1:**
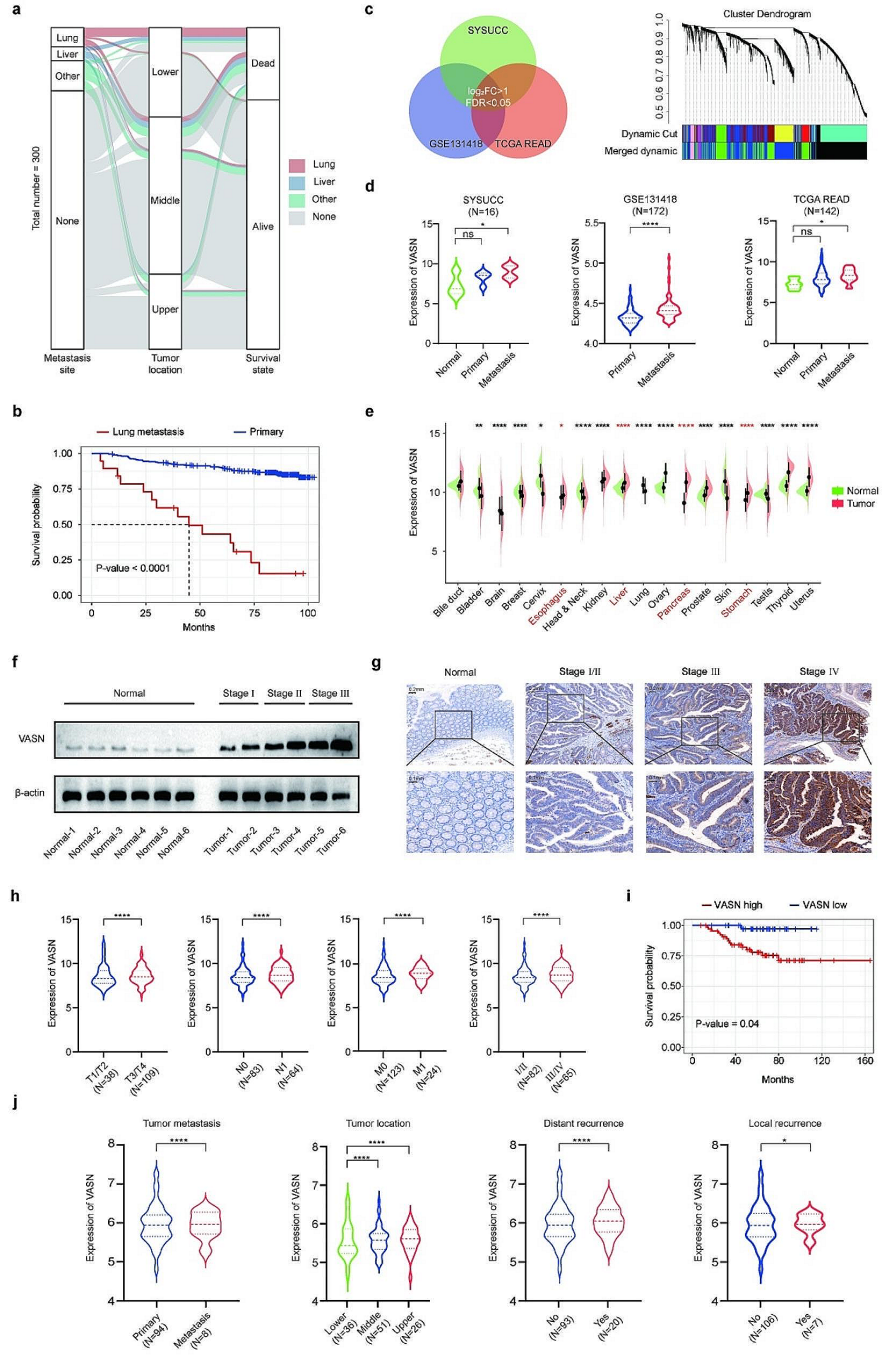
VASN expression is increased in CRC and further elevated in metastatic CRC. **a**, Clinical summary of features of 300 rectal cancer (READ) patients diagnosed at Sun Yat-sen University Cancer Center (SYSUCC) between 2015 and 2017, including metastasis site, tumor location, and survival status. **b**, OS analysis of 300 READ patients with pulmonary metastases and primary tumors. **c**, Differentially expressed gene (DEG) analysis results from three datasets and clustering patterns determined via weighted gene co-expression network (WGCNA) analysis. VASN was a common DEG among the three databases and the black clustering module. **d-e**, VASN expression in normal tissues, primary tumors, and metastatic tumors from the SYSUCC, GSE131418, TCGA rectal adenocarcinoma (TCGA-READ) and pan-cancer cohorts. **f**, Western blotting (WB) of six pairs of tumor and normal tissues from CRC patients at different clinical stages. **g**, Protein expression of VASN in CRC patients at different clinical stages determined by immunohistochemistry (IHC). **h-i**, VASN expression among patients with different TNM stages and overall survival (OS) analysis according to data obtained from the TCGA. **j**, Expression profile of VASN in patients from Memorial Sloan Kettering Cancer Center (MSKCC) with differences in metastasis status, tumor location, and recurrence status. *, P value < 0.05; **, P value < 0.01; ***, P value < 0.001

From January 1st, 2015, to January 1st, 2017, 300 patients who underwent initial rectal cancer surgery at Sun Yat-sen University Cancer Center (SYSUCC) were enrolled in this study. Analysis of the collected data revealed that middle and low rectal cancer patients had a greater incidence of pulmonary metastasis versus colon cancer (lung 6.3% vs. liver 4.6%) (Fig. 1a). Meanwhile, the five-year overall survival (OS) rate in primary patients was worse than patients with pulmonary metastasis (metastasis 37.1% vs. primary 89.8%) (Fig. 1b). To investigate the molecular mechanisms underlying pulmonary metastasis, matched pairs of rectal cancer and normal tissues from 8 patients at SYSUCC were selected for next-generation transcriptome sequencing. Differential gene expression (DEG) analysis and weighted gene co-expression network analysis (WGCNA) with a false discovery rate (FDR) < 0.05 and |fold change| (FC) > 2 were used to identify hub genes (Fig. 1c, Supplemental Fig. 1a & 1b), and data from the GSE131418 and TCGA rectal adenocarcinoma (READ) cohorts were used to validate the findings.

Vasorin (VASN) is a type I membrane protein, and its oncogenic function has been reported in various cancers. However, the underlying tumorigenic mechanism remains controversial [20–22], and its role in metastasis has not been well characterized in rectal cancer.

Our pan-cancer analysis indicated that the expression level of VASN (a specific gene of interest) was elevated in patients with pulmonary metastasis (Fig. 1d), not only in colorectal cancer (CRC) but also in various gastrointestinal tumors (Fig. 1e). Further analysis of VASN expression in patients with rectal cancer at different stages revealed that VASN expression increased as the disease progressed. To validate these findings, western blotting (WB) and immunohistochemistry (IHC) were used to analyze normal tissues and tumors at various stages (Fig. 1f and g). The results revealed that patients with pulmonary metastasis had higher levels of VASN expression than did those without pulmonary metastasis, whereas the same phenomenon was observed in patients with advanced-stage disease. Additionally, analysis of the TCGA database revealed that high VASN expression was associated with a poor prognosis (Fig. 1h) and worse five-year OS (58.9% vs. 77.6%) (Fig. 1i) in CRC patients. Consistent findings were observed in the analysis of the Memorial Sloan Kettering Cancer Center (MSKCC) dataset, which showed that high VASN expression was associated with postoperative recurrence and lower tumor location (Fig. 1j).

### VASN promotes CRC cell migration, invasion, proliferation, and drug resistance in vitro

**Fig. 2:**
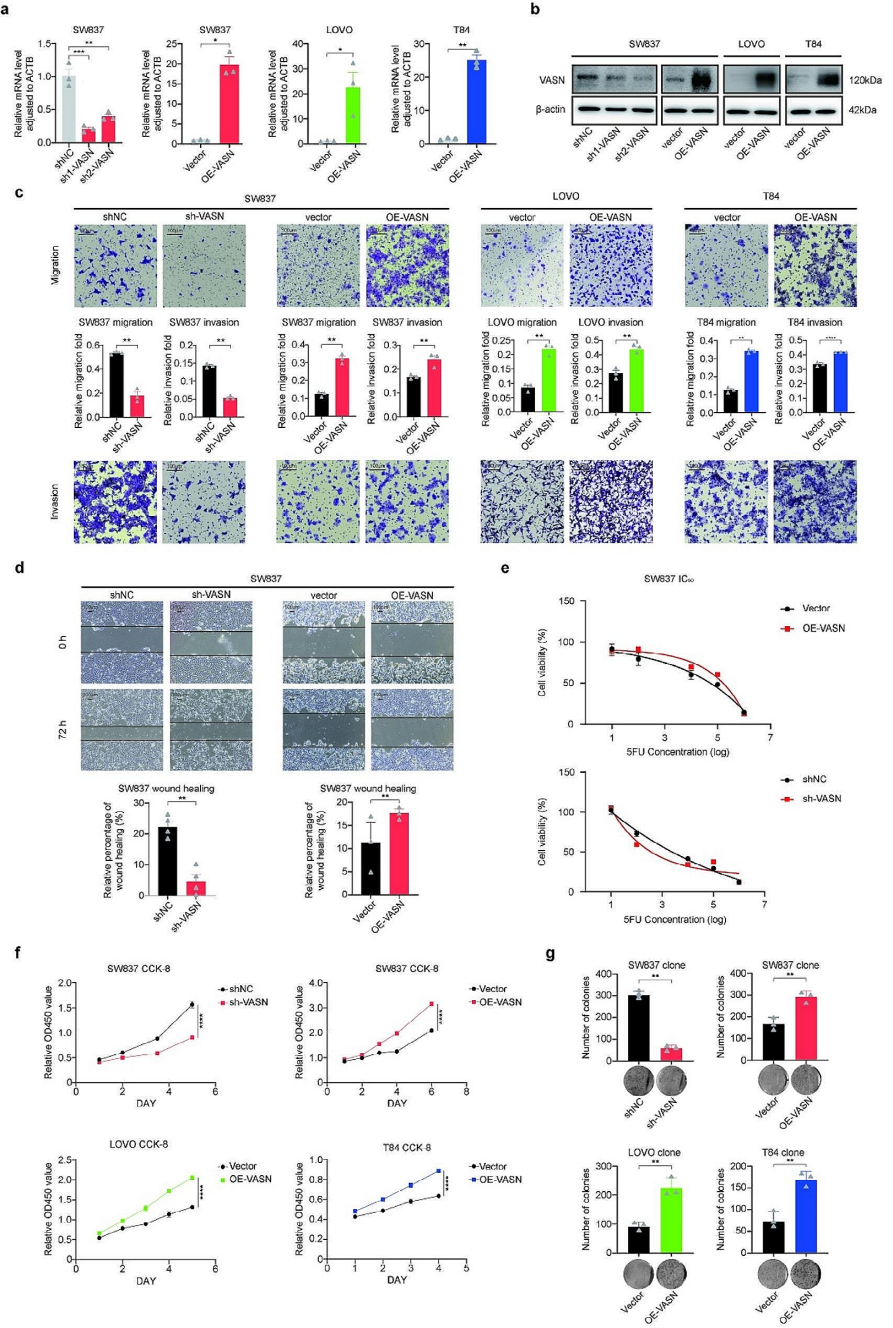
The oncogene VASN provides a survival advantage to CRC cells. **a-b**, qRT‒PCR and WB were used to validate the efficacy of VASN intervention (in vitro) in CRC cells. SW837 is a rectal cancer cell line, while LOVO and T84 cells are colon cancer cell lines. **c-d**, Transwell and wound healing assays showed that VASN promoted the migratory and invasive capabilities of CRC cells. **e**, The sensitivity to 5-fluorouracil (5-FU) was negatively correlated with VASN expression in CRC cells. **f-g**, CCK-8 and colony formation assays showed that VASN enhances the proliferation of CRC cells. All image results were quantified using bar charts for statistical comparison. *, P value < 0.05; **, P value < 0.01; ***, P value < 0.001

The levels of VASN expression in different CRC cell lines were investigated. The rectal cancer cell line SW837 exhibited higher expression of VASN than did the colon cancer cell lines LOVO and T84 at both the RNA and protein levels (Supplemental Fig. 1c). These findings were consistent with the data downloaded from the DepMap database (Supplemental Fig. 1d), which further validated our observations. To further explore the role of VASN in CRC, stable cell lines with overexpression or knockdown of VASN were generated, and the expression of VASN at the RNA and protein levels was validated (Fig. 2a and b, Supplemental Fig. 1e). Transwell assays showed that the overexpression of VASN in CRC cell lines enhanced migration and invasion (Fig. 2c, Supplemental Fig. 1f). Conversely, when VASN was knocked down, the migration and invasion abilities were weakened. Similar results were observed in a wound healing experiment (Fig. 2d, Supplemental Fig. 1g). The IC50 value was used to determine the sensitivity of CRC cells to 5-FU. Compared to control cell lines, the SW837 cell line overexpressing VASN exhibited decreased sensitivity to 5-FU. Conversely, after knocking down VASN expression, the SW837 cell line was more sensitive to 5-FU (Fig. 2e), and the same results were observed for both LOVO and T84 cells (Supplemental Fig. 1h). Additionally, CCK-8 assays were conducted to evaluate the effect of VASN on the proliferative capacity of CRC cells (Fig. 2f). The overexpression of VASN significantly promoted the proliferation of CRC cells, which was consistent with the results of the colony formation assays (Fig. 2g, Supplemental Fig. 1i). Western blot assays confirmed significant alterations in proliferation-related (CDK1) and migration-related proteins (N-cadherin) in CRC cells with VASN intervention (Supplemental Fig. 1j). Additionally, flow cytometry analysis showed that VASN overexpression promoted CRC cells into G2 phase and VASN knockdown induced apoptosis in SW837 treated with 5-FU (Supplemental Fig. 2a, Supplemental Fig. 2b). These results collectively suggest that VASN acts as an oncogene in CRC, promoting the proliferation and tumorigenesis of CRC cells.

### VASN promotes CRC cell tumorigenesis and metastasis in vivo

**Fig. 3:**
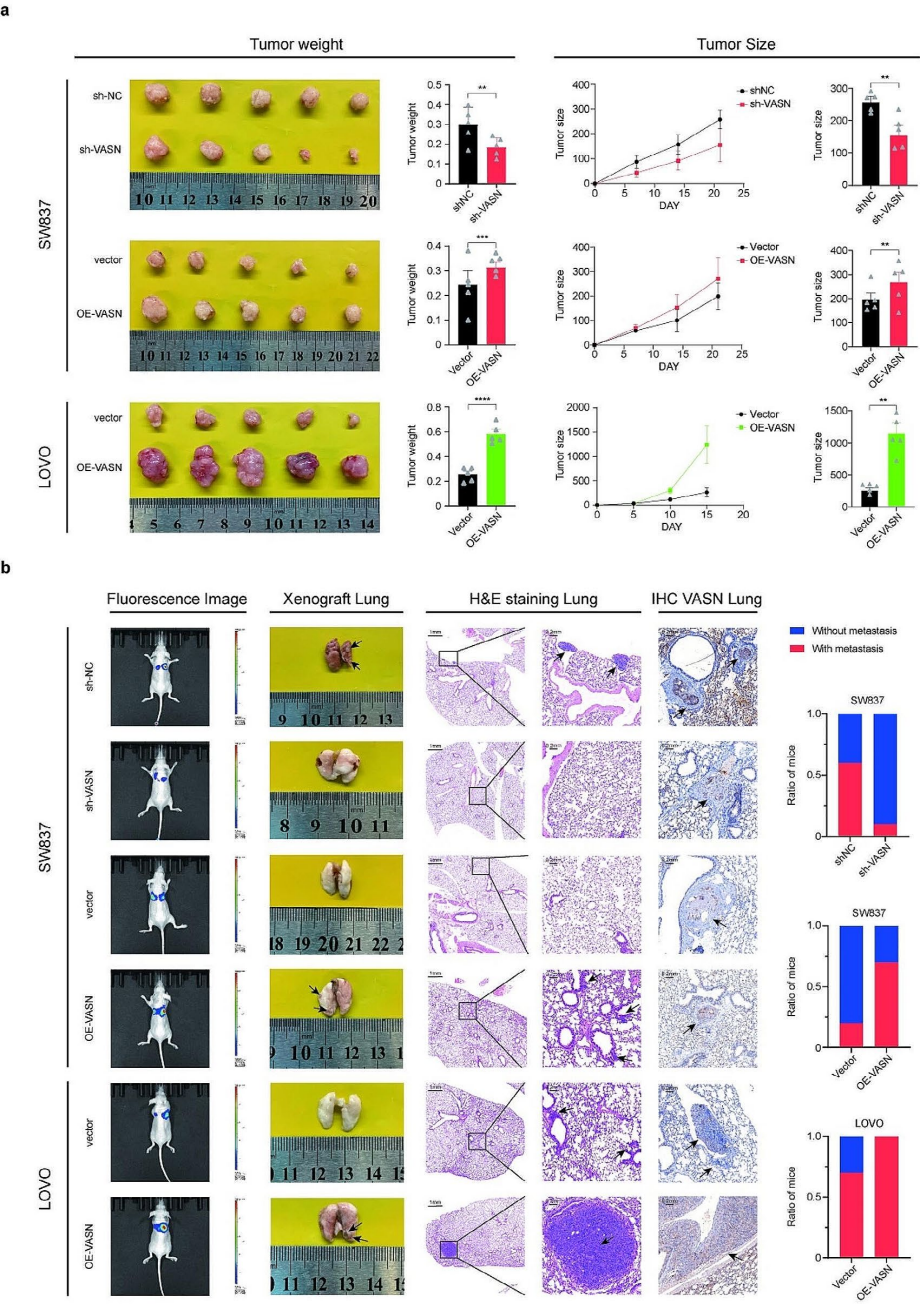
VASN promoted CRC cell tumorigenesis and pulmonary metastasis in a nude mouse model. **a-b**, BALB/c nude mice were injected with VASN-treated CRC cells (SW837 and LOVO) through flank and tail vein injection to establish subcutaneous xenograft and pulmonary metastasis models. The weight and changes in the size of the subcutaneous tumors are presented in **(a)** In the pulmonary metastasis models, luciferase and lung fluorescence images were obtained to evaluate the presence of macrometastasis. Moreover, micrometastasis was assessed by hematoxylin and eosin (HE) staining and immunohistochemistry (IHC). Images of lung metastasis and the proportions of metastatic events are displayed in **(b)** *, P value < 0.05; **, P value < 0.01; ***, P value < 0.001

To investigate whether VASN affects the growth of CRC cells in nude mice, a xenograft model was established. VASN-silenced or VASN-overexpressing CRC cells were injected into the flanks of nude mice to establish subcutaneous xenograft models. The results showed that VASN overexpression significantly increased the growth rate and tumor size, while VASN knockdown suppressed growth (Fig. 3a). Furthermore, in a nude mouse pulmonary metastasis model, a stronger bioluminescence signal and more visible metastases in the lungs were observed in the VASN-overexpressing CRC tumors (Fig. 3b columns 1 and 2). Lung hematoxylin and eosin (HE) staining of the mice revealed more and larger metastatic lesions in the CRC-overexpressing group than in the control group, and the IHC results confirmed the persistence of the VASN intervention, indicating the reliability of our experimental design (Fig. 3b columns 3 and 4). Moreover, mice injected with VASN-overexpressing cells exhibited a higher incidence of lung metastasis, while the incidence of lung metastasis was lower in mice with VASN knockdown (Fig. 3b column 5).

### The potential interaction relationship between VASN and NOTCH1

**Fig. 4:**
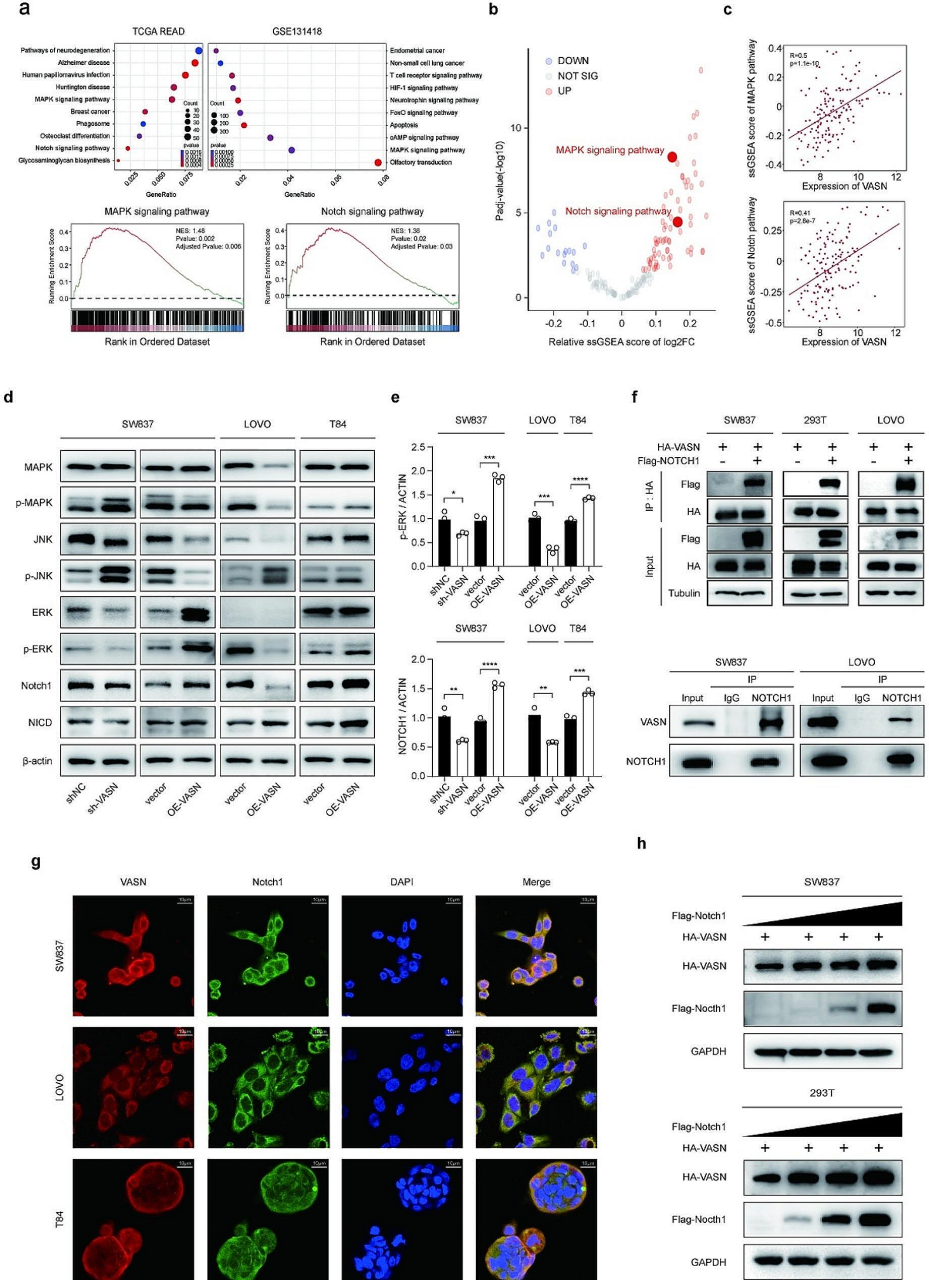
VASN regulates the MAPK and NOTCH signaling pathways.**a**, Differentially expressed genes (DEGs) from patients with high versus low VASN expression in the TCGA and GSE131418 cohorts were subjected to Gene Ontology (GO) and Kyoto Encyclopedia of Genes and Genomes (KEGG) term enrichment analysis. Among the top 10 terms, the MAPK and NOTCH pathways were enriched. **b-c**, Volcano plots displaying the correlation analysis between VASN expression and single-sample gene set enrichment analysis (ssGSEA) scores based on KEGG terms, with individual scatter plots for the MAPK pathway and NOTCH pathway. **d-e**, Western blot assays showed that VASN overexpression or knockdown in CRC cell lines altered protein expression levels within the MAPK pathway (MAPK/p-MAPK, JNK/p-JNK, ERK/p-ERK) and NOTCH (NOTCH1, NICD) pathway. **f**, Exogenous and endogenous coimmunoprecipitation (co-IP) results indicating the interaction between VASN and NOTCH1 proteins. **g**, Representative immunofluorescence (IF) images illustrating the colocalization of VASN (red) and NOTCH1 (green) in SW837, LOVO, and T84 cells. **h**, SW837 and 293T cells transfected with Flag-NOTCH1 plasmids exhibited increased endogenous VASN expression

To investigate the mechanism of action of VASN in tumorigenesis, patients were stratified based on their VASN expression levels. The DEGs were subjected to Gene Ontology (GO) and Kyoto Encyclopedia of Genes and Genomes (KEGG) enrichment analyses. By intersecting several datasets, the top 10 gene terms were ranked based on adjusted P values, which indicated enrichment of the MAPK pathway and NOTCH pathway (Fig. 4a). To enhance the reliability of our analysis, KEGG pathway gene sets from the Molecular Signatures Database (MSigDB) were downloaded, and single-sample gene set enrichment analysis (ssGSEA) was used to obtain pathway scores. The limma package was used to analyze the differences, which revealed a significant increase in the scores of both the NOTCH and MAPK pathways (Fig. 4b), and these scores were positively correlated with VASN expression (Fig. 4c). In addition, correlation analysis was performed between genes within these pathways and VASN to reveal the top 10 genes with the highest positive correlations (Supplemental Fig. 2c).

Furthermore, GeneMANIA protein data demonstrated a co-expression relationship and shared protein domains between the VASN and NOTCH1 proteins, suggesting an interaction between these two proteins (Supplemental Fig. 2d). These bioinformatics analyses collectively illustrated a potential regulatory relationship between VASN and the MAPK and NOTCH pathways. To validate this further, WB assays were performed, and the results indicated that VASN overexpression increased the expression of ERK, p-ERK, NOTCH1 and NICD (cleaved form of NOTCH1) in SW837 cells (a rectal cancer cell line), while VASN knockdown resulted in downregulation of these proteins (Fig. 4d and e, Supplemental Fig. 2e). Similar findings were observed in T84 cells (a cell line representing lung metastasis of colon cancer cells), highlighting the consistency among different cancers with pulmonary metastasis. However, inconsistent expression patterns were observed in LOVO cells (a cell lines representing lymph node metastasis of colon cancer cells), possibly due to tumor heterogeneity.

These results indicate that VASN activates both the MAPK pathway and the NOTCH pathway. To further elucidate the mechanism by which VASN regulates these pathways, coimmunoprecipitation (co-IP) experiments were performed between VASN and NOTCH1 in SW837, LOVO, and 293T cells. The results verified the interaction between VASN and NOTCH1 (Fig. 4f, Supplemental Fig. 2f), which is consistent with the prediction from GeneMANIA. Additionally, immunofluorescence (IF) staining confirmed the colocalization of endogenous VASN and NOTCH1 in the cytoplasm (Fig. 4g). Furthermore, cells were transfected with different concentrations of the Flag-NOTCH1 plasmid to observe changes in protein expression, and the results indicated that the NOTCH1 protein could increase the expression of VASN (Fig. 4h).

#### VASN regulates the MAPK pathway through the NOTCH1 protein

**Fig. 5:**
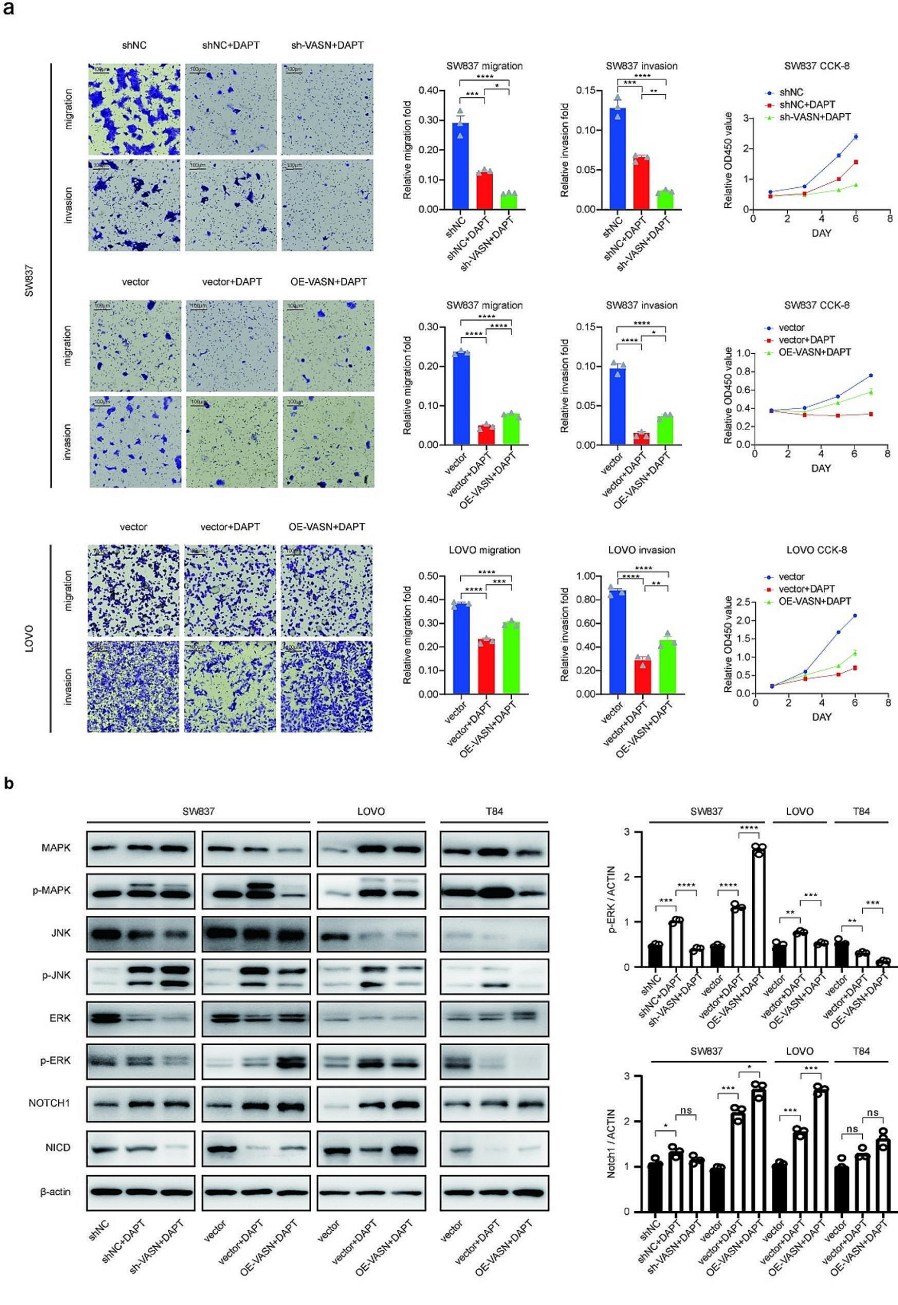
VASN overexpression partially rescued NOTCH pathway-related behaviors that were inhibited by DAPT. **a**, DAPT, a NOTCH pathway inhibitor, suppressed the migration, invasion, and proliferation of CRC cell lines. Under DAPT treatment, these behaviors were further suppressed in VASN-knockdown cells, while overexpression of VASN partially restored these behaviors. **b**, Western blot assays revealed alterations in proteins within the MAPK and NOTCH pathways in CRC cells with VASN overexpression or knockdown after DAPT treatment. *, P value < 0.05; **, P value < 0.01; ***, P value < 0.001

To investigate the relationships among the VASN, MAPK and NOTCH pathways, we used DAPT, a NOTCH pathway inhibitor. VASN-silenced cells, VASN-overexpressing cells, and negative controls were treated with DAPT. The results revealed that DAPT effectively inhibited tumor proliferation, migration, and invasion (Fig. 5a). Additionally, the effects of DAPT were markedly inhibited in VASN-silenced cells. However, when VASN was overexpressed, the effects of DAPT were partially rescued, indicating that the regulation of the phenotype by the NOTCH1 protein may be mediated through the VASN protein.

To further understand the underlying mechanisms, WB assays were conducted to analyze the expression of key components in the MAPK and NOTCH pathways. The results demonstrated that DAPT inhibits the NOTCH pathway by suppressing cleavage of the NOTCH1 (NICD) protein while concurrently partially upregulating the expression of p-MAPK, p-JNK, and p-ERK, which implies that the increase in the NOTCH1 protein level partially activates the MAPK pathway (Fig. 5b, Supplemental Fig. 2g).

Interestingly, p-ERK protein levels were slightly increased in VASN-overexpressing cells (SW837) and decreased in VASN-silenced cell lines (Fig. 5b). These results suggest that VASN may modulate the MAPK pathway through its interaction with the NOTCH1 protein and that VASN serves as a bridge that links these two crucial oncogenic pathways. The results indicated that tumor heterogeneity may be attributed to differences in protein expression between rectal cancer cells (SW837) and colon cancer cells (LOVO, T84).

### VASN expression and survival in the cohort of rectal cancer patients receiving adjuvant chemotherapy

**Fig. 6:**
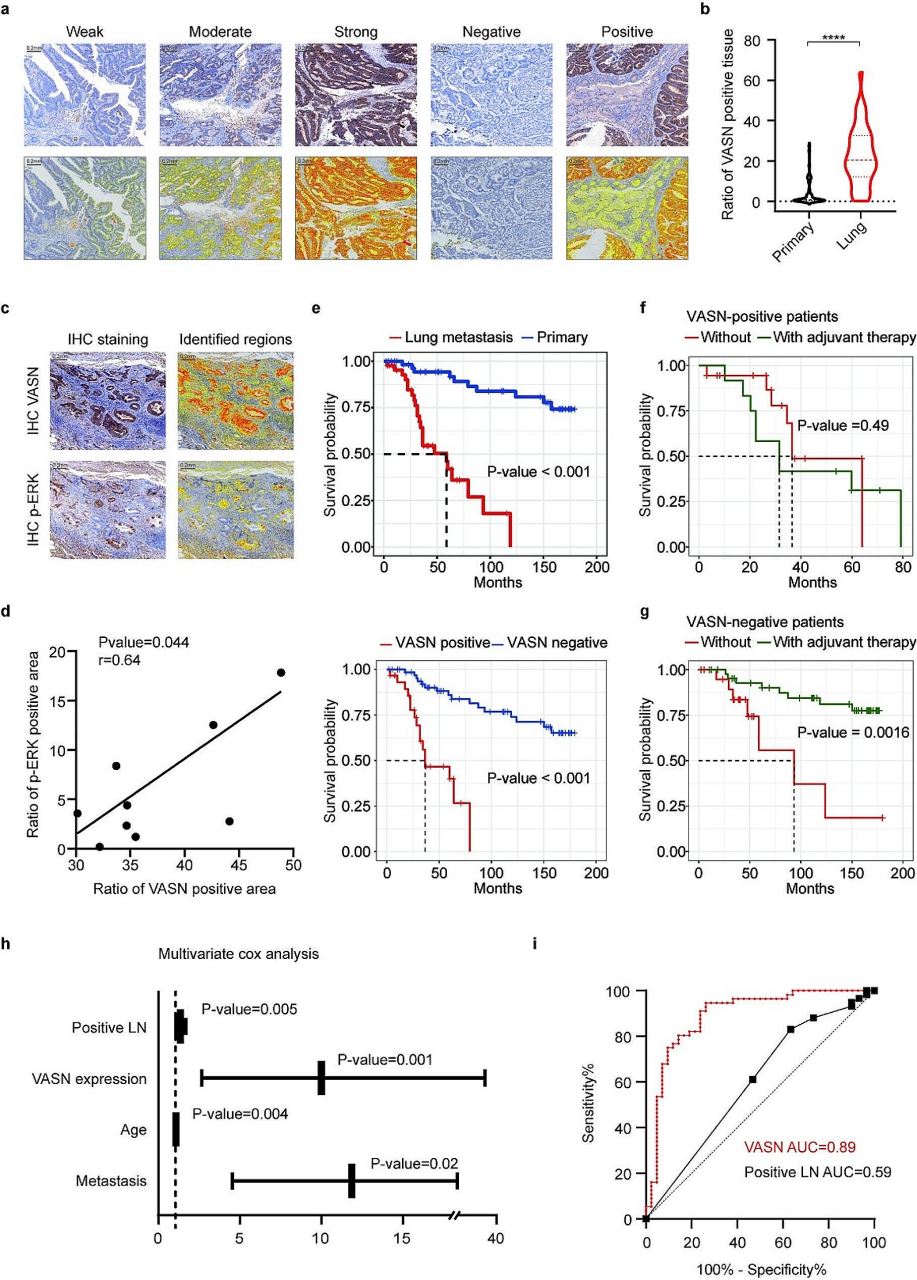
VASN overexpression is associated with pulmonary metastasis and a poor prognosis in patients. **a**, Representative immunohistochemistry (IHC) images (bright-field images and machine-identified regions) of VASN in tumor tissues, with protein expression classified as weak, moderate, strong, negative, or positive. **b**, The proportion of VASN-positive areas was greater in patients with pulmonary metastasis than in primary tumor patients. **c-d**, VASN exhibited spatial localization similar to that of p-ERK according to the IHC images (bright-field images and machine-identified regions), and the expression of these two proteins was positively correlated. **e**, In our cohort, patients with pulmonary metastasis and positive VASN expression exhibited a shorter median overall survival (OS) period. **f-g**, VASN-negative rectal cancer patients benefited from adjuvant chemotherapy more than VASN-positive patients according to the OS analysis. **h**, Multivariate Cox regression analysis was used to determine the P value and hazard regression of prognostic factors for OS. **i**, Receiver operating characteristic (ROC) curves and area under the curve (AUC) values for VASN and positive lymph nodes (positive LNs). *, P value < 0.05; **, P value < 0.01; ***, P value < 0.001

From January 1st, 2008, to January 1st, 2018, clinical information and tissue samples were collected from 98 rectal cancer patients, including 56 primary tumor patients and 42 patients with pulmonary metastasis at SYSUCC. The expression of VASN, which was detected by IHC, was used to divide patients into positive and negative groups (Fig. 6a). Among them, 30 patients (30.6%) were VASN-positive, and 68 patients (69.4%) were VASN-negative (Table 1). 90% of the VASN-positive patients exhibited pulmonary metastasis, whereas 22.1% of the patients with negative VASN staining had pulmonary metastasis. The IHC score also indicated that the proportion of VASN-positive tissue in pulmonary metastasis patients was greater than that in primary tumor patients (20.4% vs. 6.7%) (Fig. 6b). Additionally, the expression of p-ERK was also quantified according to the IHC score. The IHC results showed that VASN and p-ERK exhibited similar spatial localization (Fig. 6c), and there was a positive correlation between VASN and p-ERK scores (Fig. 6d). In our cohort, the median follow-up period of pulmonary metastasis patients was 58.83 months, and the five-year survival rate for the VASN-positive group was 26.7%, compared to 83.7% for the VASN-negative group (Fig. 6e). Although there was no significant difference, the levels of tumor markers tended to be higher in the positive group (CEA 37.1 vs. 13.2, CA199 103 vs. 24.6) (Table 1). Patients with VASN overexpression tended to have a higher risk of metastasis; however, the OS analysis results indicated that they did not benefit from adjuvant chemotherapy (Fig. 6f). On the other hand, the OS analysis revealed that VASN-negative patients benefited from adjuvant therapy (Fig. 6g), which may be explained by drug resistance, as described above.

**Table 1:** Clinical characteristics of VASN protein expression with rectal cancer patients from SYSUCC cohort

	VASN-negative	VASN-positive	*P* value
	(*N* = 68)	(*N* = 30)	
**Age**			
Mean (SD)	55.0 (12.3)	53.9 (8.48)	0.591
Median [Min, Max]	56.0 [27.0, 75.0]	56.5 [32.0, 68.0]	
**Sex**			
Female	27 (39.7%)	10 (33.3%)	0.709
Male	41 (60.3%)	20 (66.7%)	
**BMI**			
Mean (SD)	22.8 (3.19)	23.6 (3.02)	0.285
Median [Min, Max]	22.6 [16.9, 30.3]	23.1 [18.6, 33.7]	
Missing	1 (1.5%)	0 (0%)	
**Smoking**			
No	46 (67.6%)	23 (76.7%)	0.508
Yes	22 (32.4%)	7 (23.3%)	
**pN stage**			
Mean (SD)	0.585 (0.659)	0.545 (0.739)	0.827
Median [Min, Max]	0 [0, 2.00]	0 [0, 2.00]	
Missing	3 (4.4%)	8 (26.7%)	
**positive LN**			
Mean (SD)	1.48 (3.19)	0.750 (1.33)	0.147
Median [Min, Max]	0 [0, 22.0]	0 [0, 5.00]	
Missing	3 (4.4%)	10 (33.3%)	
**MRF**			
Negative	5 (7.4%)	5 (16.7%)	
Positive	4 (5.9%)	5 (16.7%)	1
Missing	59 (86.8%)	20 (66.7%)	
**EMVI**			
Negative	4 (5.9%)	4 (13.3%)	
Positive	5 (7.4%)	3 (10.0%)	1
Missing	59 (86.8%)	23 (76.7%)	
**CRM**			
Negative	61 (89.7%)	16 (53.3%)	
Positive	0 (0%)	1 (3.3%)	0.492
Missing	7 (10.3%)	13 (43.3%)	
**CEA**			
Mean (SD)	13.2 (28.2)	37.1 (159)	0.427
Median [Min, Max]	3.50 [0.400, 170]	2.54 [0.613, 853]	
Missing	1 (1.5%)	1 (3.3%)	
**CA199**			
Mean (SD)	24.6 (29.3)	103 (449)	0.384
Median [Min, Max]	14.3 [0.600, 181]	12.9 [0.600, 2300]	
Missing	2 (2.9%)	4 (13.3%)	
**Neoadjuvant**			
No	47 (69.1%)	16 (53.3%)	0.198
Yes	19 (27.9%)	13 (43.3%)	
Missing	2 (2.9%)	1 (3.3%)	
**Neoadjuvant** **Chemotherapy**			
No	49 (72.1%)	18 (60.0%)	0.0065
Standard	7 (10.3%)	0 (0%)	
Long	8 (11.8%)	11 (36.7%)	
Missing	4 (5.9%)	1 (3.3%)	
**Neoadjuvant** **Radiotherapy**			
No	56 (82.4%)	19 (63.3%)	0.0636
Yes	10 (14.7%)	10 (33.3%)	
Missing	2 (2.9%)	1 (3.3%)	
**Adjuvant chemotherapy**			
No	24 (35.3%)	18 (60.0%)	0.0398
Yes	44 (64.7%)	12 (40.0%)	
**Metastasis**			
Primary	53 (77.9%)	3 (10.0%)	
Synchronous	10 (14.7%)	12 (40.0%)	< 0.001
metachronous	5 (7.4%)	15 (50.0%)	
**OS outcome**			
Alive	52 (76.5%)	14 (46.7%)	0.00768
Dead	16 (23.5%)	16 (53.3%)	
**Follow-up time**			
Mean (SD)	96.0 (61.8)	34.7 (21.1)	< 0.001
Median [Min, Max]	90.3 [2.03, 180]	31.5 [3.07, 79.2]	
**Distance to anus**			
Low-middle	42 (61.8%)	13 (43.3%)	0.963
Upper	22 (32.4%)	8 (26.7%)	
Missing	4 (5.9%)	9 (30.0%)	

In the univariate regression analysis, VASN group (positive vs. negative; HR = 7.4, P value < 0.001) and pulmonary metastasis status (HR = 11.88, P value < 0.001) showed a strong relationship with OS, and in the multivariate regression analysis, VASN group (HR = 10.01, P value = 0.001) remained a significant prognostic factor for OS (Fig. 6h; Table 2). Similarly, compared with the area under the curve (AUC) for positive lymph nodes (positive LNs), the AUC for VASN expression was greater according to receiver operating characteristic (ROC) curve analysis (0.89 vs. 0.59) (Fig. 6i). These findings indicate that VASN expression is associated with pulmonary metastasis and a poor prognosis and might be associated with drug resistance; furthermore, it can serve as an independent risk factor for OS in rectal cancer patients.

**Table 2:** Univariate and multivariate survival analysis of rectal cancer patients in SYSUCC cohort

		Overall survival (OS)			
Variables		Univariate		Multivariate	
	*N* (%)	HR (95% CI)	*P* value	HR (95% CI)	*P* value
**Age**					
Mean ± SD	54.2 ± 11.6	1.03 (0.99–1.06)	0.101	1.08 (1.03–1.14)	0.004
**Sex**					
Male	31 (38.8%)				
Female	49 (61.2%)	1.93 (0.87–4.29)	0.108	2.42 (0.71–8.24, *p* = .159)	0.159
**BMI**					
Mean ± SD	22.8 ± 3.1	0.89 (0.80–1.01)	0.061	0.88 (0.74–1.04, *p* = .145)	0.145
**Smoking**					
No	56 (70.0%)				
Yes	24 (30.0%)	1.70 (0.84–3.42)	0.137	0.49 (0.18–1.29, *p* = .148)	0.148
**pN stage**					
N0	44 (55.0%)				
N1	28 (35.0%)				
N2	8 (10.0%)	1.35 (0.45–4.07)	0.595		
**positive LN**	1.3 ± 3.0	1.12 (0.95–1.33)	0.16	1.36 (1.09–1.68, *p* = .005)	0.005
**CEA**					
Mean ± SD	12.3 ± 28.9	1.01 (0.99–1.02)	0.329		
**CA199**					
Mean ± SD	21.8 ± 27.3	1.00 (0.98–1.01)	0.7		
**Neoadjuvant**					
No	55 (68.8%)				
Yes	25 (31.2%)	2.51 (1.22–5.16)	0.012	1.12 (0.47–2.65, *p* = .803)	0.803
**Adjuvant chemotherapy**					
No	29 (36.2%)				
Yes	51 (63.8%)	0.39 (0.18–0.81)	0.011	1.35 (0.50–3.68, *p* = .552)	0.552
**Metastasis**					
Primary	56 (57.1%)				
Metastasis	42 (42.9%)	11.88 (4.55–31.05)	< 0.001	4.38 (1.27–15.15, *p* = .020)	0.02
**Distance to anus**					
Low-Middle	53 (66.2%)				
High(> 10 cm)	27 (33.8%)	1.13 (0.52–2.46)	0.755		
**VASN group**					
Negative	62 (77.5%)				
Positive	18 (22.5%)	7.42 (3.22–17.07)	< 0.001	10.01 (2.67–37.45, *p* = .001)	0.001

## Discussion

Rectal cancer patients are more likely to have pulmonary metastasis than colon cancer patients are, possibly because of differences in embryonic development and blood supply [13, 27, 28]. Global epidemiological data on colorectal cancer also indicate that rectal cancer patients are more likely to experience pulmonary metastasis [14, 29]. However, the molecular mechanism of pulmonary metastasis is still unknown. Adjuvant therapy has been proven to eliminate micrometastases and improve OS in CRC patients [6–9], but rectal cancer patients benefit less than colon cancer patients [30]. The differences in molecular subtypes may explain this disparity, which highlights the need for improvement in the precision of patient stratification for treatment selection [16]. Our study revealed for the first time the potential molecular mechanisms of pulmonary metastasis and adjuvant chemotherapy failure in patients with rectal cancer. These findings suggest that the VASN may serve as a valuable tissue-based risk indicator for pulmonary metastasis events and a predictive factor for adjuvant therapy efficacy.

The processes of invasion and metastasis, which are hallmarks of cancer, involve a complex cascade of events [31]. Our study revealed a significant correlation between VASN expression and pulmonary metastasis events and a poor prognosis in CRC patients, particularly in mid-low rectal cancer cases. In addition, high VASN expression was associated with a lower rectal cancer site, a higher incidence of recurrence and metastasis, and worse overall survival (30.8% five-year survival rate). Our results also showed that VASN expression promoted the proliferation, drug resistance, and metastasis of CRC cells. In a nude mouse pulmonary metastasis model, VASN facilitated the development of lung-metastatic tumors.

The underlying mechanism by which VASN influences tumor progression was also investigated. Experiments confirmed the interaction between the VASN and NOTCH1 proteins, highlighting the coregulation of the NOTCH1 pathway and MAPK pathway. Furthermore, rescue experiments revealed that the VASN protein modulates the MAPK pathway through interaction with NOTCH1, revealing a novel regulatory mechanism for VASN-mediated MAPK pathway activation. Additionally, this study showed that patients with positive VASN expression had a greater incidence of pulmonary metastasis. A correlation between VASN and p-ERK protein expression in tumor tissues was also observed, further confirming the activation of the MAPK pathway by VASN in tumor tissues.

Tumors are complex diseases, and the spreading of tumor cells to other parts of the body is known as metastasis [32–34]. When tumor cells detach from the primary site, they often exhibit certain characteristics, such as reduced cell adhesion and increased migratory and invasive abilities. These characteristics are often associated with the activation of the WNT signaling pathway [33, 35, 36]. Previous studies have suggested that VASN, a protein associated with vascular function, primarily influences vascular function through the TGFβ signaling pathway [37], and a recent study on breast cancer yielded a similar conclusion [38]. Although there are numerous studies on VASN function in tumorigenesis, the molecular mechanisms involved in metastasis have not been fully elucidated. Our study suggested that VASN activates the NOTCH pathway [24] and regulates the MAPK pathway by binding to the NOTCH1 protein. The MAPK and NOTCH pathways are critical oncogenic pathways in tumorigenesis, and VASN acts as a bridge to simultaneously activate both pathways. This provides valuable molecular insights into the mechanisms underlying metastasis.

Adjuvant chemotherapy prevents tumor recurrence by targeting micrometastases, but its use and efficacy in rectal cancer patients remain controversial. Treatment selection based on tumor molecular subtypes may increase patient benefit; however, current guidelines do not consider any predictive biomarkers for adjuvant therapy [10, 39]. In our study, we found that rectal cancer patients with pulmonary metastasis had higher expression of VASN, which is typically associated with advanced TNM stage. Additionally, in our patient cohort, patients with high VASN expression experienced more frequent pulmonary metastasis events and had shorter five-year OS. According to univariate regression analysis and multivariate regression analysis, there was a significant correlation between VASN expression and short-term survival (HR = 7.9, P value < 0.01).

These findings suggest that targeting VASN and its associated pathways might be a promising therapeutic strategy for rectal cancer patients. However, further studies and clinical trials are needed to validate these findings and explore the potential benefits of targeted therapy in VASN-positive patients. By designing personalized treatment plans based on VASN expression, we aim to improve patient outcomes and ultimately improve the management of rectal cancer. In our study, LARC patients from SYSUCC cohort had a wide range of diagnosis and treatment timelines, and there were variations in the treatment approaches for patients in different time periods. Furthermore, the treatment process for metastatic patients is complex, often involving multiple hospitals, all of which poses challenges for our conclusion.

Although rectal cancer patients without high-risk factors currently do not require adjuvant therapy, our findings suggest that VASN-negative patients should receive adjuvant chemotherapy, as this approach may improve survival. This recommendation underscores the importance of targeting VASN and its associated pathways as a promising therapeutic approach for managing rectal cancer. However, further studies and clinical trials are necessary to validate our findings and explore the potential benefits of targeted therapies for VASN-positive patients.

## Conclusions

**Fig. 7:**
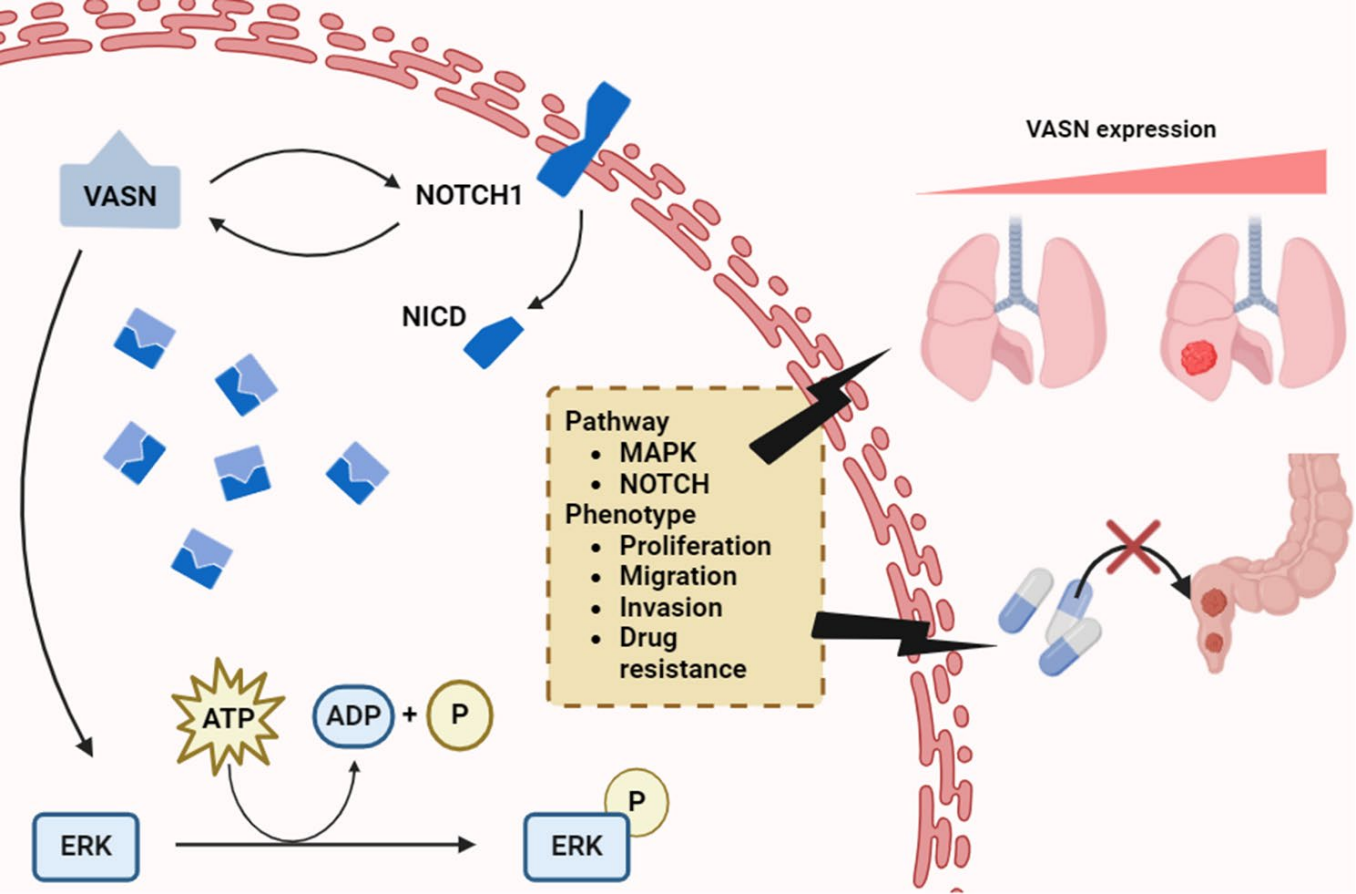
Schematic diagram of VASN function in CRC. VASN promotes pulmonary metastasis and chemotherapy resistance through the activation of the MAPK and NOTCH pathways in LARC patients

In conclusion, our research confirmed for the first time that VASN acts as an oncogene, promoting pulmonary metastasis through the activation of the MAPK and NOTCH pathways in rectal cancer. Furthermore, a clinical cohort analysis revealed specific characteristics of VASN-positive patients. These findings support the potential value of VASN as a biomarker for assessing metastatic risk in rectal cancer patients (Fig. 7).

### Electronic supplementary material

Below is the link to the electronic supplementary material.

Supplementary Material 1

Supplementary Material 2

## Data Availability

All authors had access to the data published in this paper. Data have been uploaded to the Research Data online platform (https://www.researchdata.org.cn/).

## References

[1] Bray F, Ferlay J, Soerjomataram I, Siegel RL, Torre LA, Jemal A. Global cancer statistics 2018: GLOBOCAN estimates of incidence and mortality worldwide for 36 cancers in 185 countries. Cancer J Clin. 2018;68(6):394–424. 10.3322/caac.21492. 10.3322/caac.21492 30207593

[2] Peacock O, Waters PS, Bressel M, Lynch AC, Wakeman C, Eglinton T, et al. Prognostic factors and patterns of failure after surgery for T4 rectal cancer in the beyond total mesorectal excision era. Br J Surg. 2019;106(12):1685–96. 10.1002/bjs.11242. 31339561 10.1002/bjs.11242

[3] Bosset J-F, Collette L, Calais G, Mineur L, Maingon P, Radosevic-Jelic L, et al. Chemotherapy with preoperative radiotherapy in rectal cancer. N Engl J Med. 2006;355(11):1114–23. 16971718 10.1056/NEJMoa060829

[4] Sainato A, Cernusco Luna Nunzia V, Valentini V, De Paoli A, Maurizi ER, Lupattelli M, et al. No benefit of adjuvant Fluorouracil Leucovorin chemotherapy after neoadjuvant chemoradiotherapy in locally advanced cancer of the rectum (LARC): long term results of a randomized trial (I-CNR-RT). Radiother Oncol. 2014;113(2):223–9. 10.1016/j.radonc.2014.10.006. 25454175 10.1016/j.radonc.2014.10.006

[5] Breugom AJ, van Gijn W, Muller EW, Berglund A, van den Broek CBM, Fokstuen T, et al. Adjuvant chemotherapy for rectal cancer patients treated with preoperative (chemo)radiotherapy and total mesorectal excision: a Dutch Colorectal Cancer Group (DCCG) randomized phase III trial. Ann Oncol. 2015;26(4):696–701. 10.1093/annonc/mdu560. 25480874 10.1093/annonc/mdu560

[6] Quasar Collaborative G, Gray R, Barnwell J, McConkey C, Hills RK, Williams NS, et al. Adjuvant chemotherapy versus observation in patients with colorectal cancer: a randomised study. Lancet. 2007;370(9604):2020–9. 10.1016/S0140-6736(07)61866-2. 18083404 10.1016/S0140-6736(07)61866-2

[7] Andre T, de Gramont A, Vernerey D, Chibaudel B, Bonnetain F, Tijeras-Raballand A, et al. Adjuvant fluorouracil, leucovorin, and oxaliplatin in stage II to III Colon cancer: updated 10-Year survival and outcomes according to BRAF Mutation and Mismatch Repair Status of the MOSAIC Study. J Clin Oncol. 2015;33(35):4176–87. 10.1200/JCO.2015.63.4238. 26527776 10.1200/JCO.2015.63.4238

[8] Haller DG, Tabernero J, Maroun J, de Braud F, Price T, Van Cutsem E, et al. Capecitabine plus oxaliplatin compared with fluorouracil and folinic acid as adjuvant therapy for stage III colon cancer. J Clin Oncol. 2011;29(11):1465–71. 10.1200/JCO.2010.33.6297. 21383294 10.1200/JCO.2010.33.6297

[9] Andre T, Meyerhardt J, Iveson T, Sobrero A, Yoshino T, Souglakos I, et al. Effect of duration of adjuvant chemotherapy for patients with stage III colon cancer (IDEA collaboration): final results from a prospective, pooled analysis of six randomised, phase 3 trials. Lancet Oncol. 2020;21(12):1620–9. 10.1016/S1470-2045(20)30527-1. 33271092 10.1016/S1470-2045(20)30527-1 PMC7786835

[10] Chaffer CL, Weinberg RA. A perspective on Cancer Cell Metastasis. Science. 2011;331(6024):1559–64. 10.1126/science.1203543. 21436443 10.1126/science.1203543

[11] Kosmider S. Lara Lipton. Adjuvant therapies for colorectal cancer. World J Gastroenterol 2007; 28;13(28):3799 – 805 10.3748/wjg.v13.i28.3799. 10.3748/wjg.v13.i28.3799 PMC4611211 17657833

[12] Bhullar DS, Barriuso J, Mullamitha S, Saunders MP, O’Dwyer ST, Aziz O. Biomarker concordance between primary colorectal cancer and its metastases. EBioMedicine. 2019;40:363–74. 10.1016/j.ebiom.2019.01.050. 30733075 10.1016/j.ebiom.2019.01.050 PMC6413540

[13] Riihimaki M, Hemminki A, Sundquist J, Hemminki K. Patterns of metastasis in colon and rectal cancer. Sci Rep. 2016;6:29765. 10.1038/srep29765. 27416752 10.1038/srep29765 PMC4945942

[14] Siegel RL, Miller KD, Fuchs HE, Jemal A. Cancer statistics, 2022. Cancer J Clin. 2022;72(1):7–33. 10.3322/caac.21708. 10.3322/caac.21708 35020204

[15] Ge Y, Lei S, Cai B, Gao X, Wang G, Wang L, et al. Incidence and prognosis of pulmonary metastasis in colorectal cancer: a population-based study. Int J Colorectal Dis. 2020;35:223–32. 31823051 10.1007/s00384-019-03434-8

[16] Yang L, Yang J, Kleppe A, Danielsen HE, Kerr DJ. Personalizing adjuvant therapy for patients with colorectal cancer. Nat Rev Clin Oncol. 2023. 10.1038/s41571-023-00834-2. 38001356 10.1038/s41571-023-00834-2

[17] Schostak M, Bradbury A, Briganti A, Gonzalez D, Gomella L, Mateo J, et al. Practical Guidance on establishing a molecular testing pathway for alterations in homologous recombination repair genes in clinical practice for patients with metastatic prostate Cancer. Eur Urol Oncol. 2023. 10.1016/j.euo.2023.08.004. 37714762 10.1016/j.euo.2023.08.004

[18] Foersch S, Glasner C, Woerl AC, Eckstein M, Wagner DC, Schulz S, et al. Multistain deep learning for prediction of prognosis and therapy response in colorectal cancer. Nat Med. 2023;29(2):430–9. 10.1038/s41591-022-02134-1. 36624314 10.1038/s41591-022-02134-1

[19] Pages F, Mlecnik B, Marliot F, Bindea G, Ou FS, Bifulco C, et al. International validation of the consensus immunoscore for the classification of colon cancer: a prognostic and accuracy study. Lancet. 2018;391(10135):2128–39. 10.1016/S0140-6736(18)30789-X. 29754777 10.1016/S0140-6736(18)30789-X

[20] Louvet L, Lenglet G, Krautzberger AM, Mentaverri R, Hague F, Kowalewski C, et al. Vasorin plays a critical role in vascular smooth muscle cells and arterial functions. J Cell Physiol. 2022;237(10):3845–59. 10.1002/jcp.30838. 35892191 10.1002/jcp.30838 PMC9796581

[21] Chen L, Yao JH, Zhang SH, Wang L, Song HD, Xue JL. Slit-like 2, a novel zebrafish slit homologue that might involve in zebrafish central neural and vascular morphogenesis. Biochem Biophys Res Commun. 2005;336(1):364–71. 10.1016/j.bbrc.2005.08.071. 16125671 10.1016/j.bbrc.2005.08.071

[22] Krautzberger AM, Kosiol B, Scholze M, Schrewe H. Expression of vasorin (Vasn) during embryonic development of the mouse. Gene Expr Patterns. 2012;12(5–6):167–71. 10.1016/j.gep.2012.02.003. 22426063 10.1016/j.gep.2012.02.003

[23] Malapeira J, Esselens C, Bech-Serra JJ, Canals F, Arribas J. ADAM17 (TACE) regulates TGFbeta signaling through the cleavage of vasorin. Oncogene. 2011;30(16):1912–22. 10.1038/onc.2010.565. 21170088 10.1038/onc.2010.565

[24] Liang W, Guo B, Ye J, Liu H, Deng W, Lin C, et al. Vasorin stimulates malignant progression and angiogenesis in glioma. Cancer Sci. 2019;110(8):2558–72. 10.1111/cas.14103. 31215106 10.1111/cas.14103 PMC6676100

[25] Deng Y, Chi P, Lan P, Wang L, Chen W, Cui L, et al. Modified FOLFOX6 with or without radiation versus fluorouracil and leucovorin with radiation in neoadjuvant treatment of locally advanced rectal cancer: initial results of the Chinese FOWARC multicenter, open-label, randomized three-arm phase III trial. J Clin Oncol. 2016;34(27):3300–7. 27480145 10.1200/JCO.2016.66.6198

[26] Guo X, Chen F, Gao F, Li L, Liu K, You L et al. CNSA: a data repository for archiving omics data. Database 2020;2020. 10.1093/database/baaa055 PMC7377928 32705130

[27] Tamas K, Walenkamp AM, de Vries EG, van Vugt MA, Beets-Tan RG, van Etten B, et al. Rectal and colon cancer: not just a different anatomic site. Cancer Treat Rev. 2015;41(8):671–9. 10.1016/j.ctrv.2015.06.007. 26145760 10.1016/j.ctrv.2015.06.007

[28] Paschke S, Jafarov S, Staib L, Kreuser ED, Maulbecker-Armstrong C, Roitman M, et al. Are Colon and rectal Cancer two different tumor entities? A proposal to abandon the term colorectal Cancer. Int J Mol Sci. 2018;19(9). 10.3390/ijms19092577. 10.3390/ijms19092577 PMC6165083 30200215

[29] Xia C, Dong X, Li H, Cao M, Sun D, He S, et al. Cancer statistics in China and United States, 2022: profiles, trends, and determinants. Chin Med J (Engl). 2022;135(5):584–90. 10.1097/CM9.0000000000002108. 35143424 10.1097/CM9.0000000000002108 PMC8920425

[30] Song N, Pogue-Geile KL, Gavin PG, Yothers G, Kim SR, Johnson NL, et al. Clinical outcome from oxaliplatin treatment in stage II/III Colon cancer according to intrinsic subtypes: secondary analysis of NSABP C-07/NRG oncology Randomized Clinical Trial. JAMA Oncol. 2016;2(9):1162–9. 10.1001/jamaoncol.2016.2314. 27270348 10.1001/jamaoncol.2016.2314 PMC5065181

[31] Hanahan D. Hallmarks of Cancer: New dimensions. Cancer Discov. 2022;12(1):31–46. 10.1158/2159-8290.CD-21-1059. 35022204 10.1158/2159-8290.CD-21-1059

[32] Gupta GP, Massagué J. Cancer Metastasis: building a Framework. Cell. 2006;127(4):679–95. 10.1016/j.cell.2006.11.001. 17110329 10.1016/j.cell.2006.11.001

[33] Allgayer H, Leupold JH, Patil N. Defining the Metastasome: perspectives from the genome and molecular landscape in colorectal cancer for metastasis evolution and clinical consequences. Sem Cancer Biol. 2020;60:1–13. 10.1016/j.semcancer.2019.07.018. 10.1016/j.semcancer.2019.07.018 31362074

[34] Klein CA. Cancer progression and the invisible phase of metastatic colonization. Nat Rev Cancer. 2020;20(11):681–94. 10.1038/s41568-020-00300-6. 33024261 10.1038/s41568-020-00300-6

[35] Delattre J-F, Selcen Oguz Erdogan A, Cohen R, Shi Q, Emile J-F, Taieb J, et al. A comprehensive overview of tumour deposits in colorectal cancer: towards a next TNM classification. Cancer Treat Rev. 2022;103. 10.1016/j.ctrv.2021.102325. 10.1016/j.ctrv.2021.102325 34954486

[36] Lugli A, Zlobec I, Berger MD, Kirsch R, Nagtegaal ID. Tumour budding in solid cancers. Nat Rev Clin Oncol. 2021;18(2):101–15. 10.1038/s41571-020-0422-y. 32901132 10.1038/s41571-020-0422-y

[37] Luong TTD, Estepa M, Boehme B, Pieske B, Lang F, Eckardt KU, et al. Inhibition of vascular smooth muscle cell calcification by vasorin through interference with TGFbeta1 signaling. Cell Signal. 2019;64:109414. 10.1016/j.cellsig.2019.109414. 31505229 10.1016/j.cellsig.2019.109414

[38] Yeo HL, Fan TC, Lin RJ, Yu JC, Liao GS, Chen ES, et al. Sialylation of vasorin by ST3Gal1 facilitates TGF-beta1-mediated tumor angiogenesis and progression. Int J Cancer. 2019;144(8):1996–2007. 10.1002/ijc.31891. 30252131 10.1002/ijc.31891 PMC6590135

[39] Glynne-Jones R, Wyrwicz L, Tiret E, Brown G, Rodel C, Cervantes A, et al. Rectal cancer: ESMO Clinical Practice guidelines for diagnosis, treatment and follow-up. Ann Oncol. 2017;28(suppl4):iv22–40. 10.1093/annonc/mdx224. 28881920 10.1093/annonc/mdx224

