# Supplementary material for: Vasorin (VASN) overexpression promotes pulmonary metastasis and resistance to adjuvant chemotherapy in patients with locally advanced rectal cancer
Source: J Transl Med. 2024 Aug 6;22:742. doi: 10.1186/s12967-024-05473-4 (PMC11301854; doi:10.1186/s12967-024-05473-4)
Supplement: Supplementary file 1 — Supplementary Material 1 [file 12967_2024_5473_MOESM1_ESM.docx]

**Supplementary Figure 1**


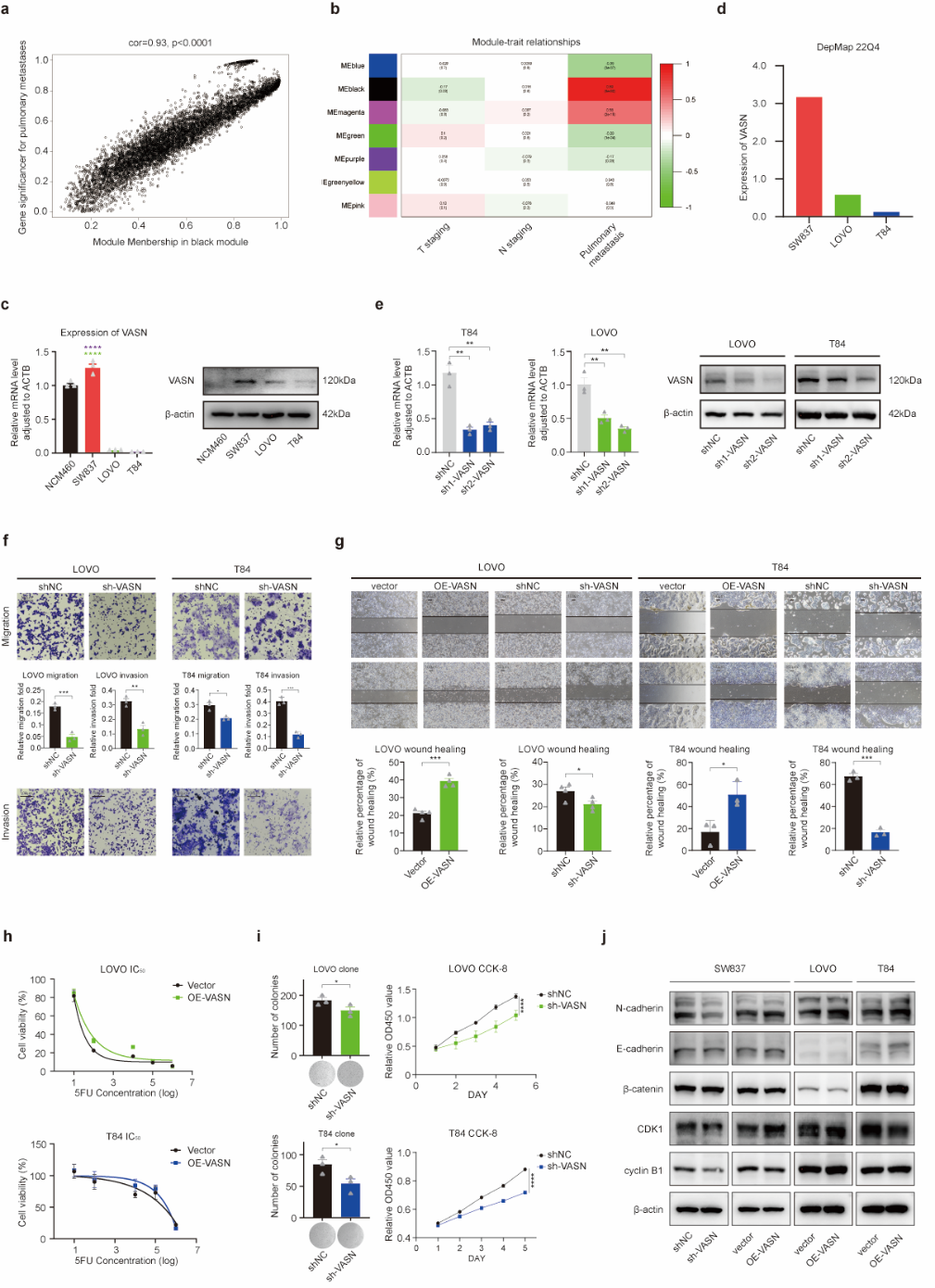


**a-b,** Weighted gene co-expression network analysis (WGCNA) revealed a robust positive correlation between the black module and pulmonary metastasis events, with a strong correlation in gene expression within the module (r=0.93, P-value<0.0001). **c,** qRT‒PCR and WB were used to evaluate the expression levels of VASN in experimental CRC cell lines. **d,** The expression level of VASN downloaded from the DepMap (22Q4) dataset in experimental CRC cell lines. **e,** qRT‒PCR and WB were used to validate the efficacy of VASN intervention in LOVO and T84 cells. **f-g,** VASN promoted migration and reduced drug sensitivity in LOVO and T84 cells. **h,** The assessment of sensitivity to 5-FU in LOVO and T84 cells. **i,** CCK-8 and colony formation assays in LOVO and T84 cells. **j,** Western blot assays demonstrated the expression of proliferative and migrative proteins altered in CRC cells with VASN overexpression or knockdown. *, P value < 0.05; **, P value < 0.01; ***, P value < 0.001.

**Supplementary Figure 2**


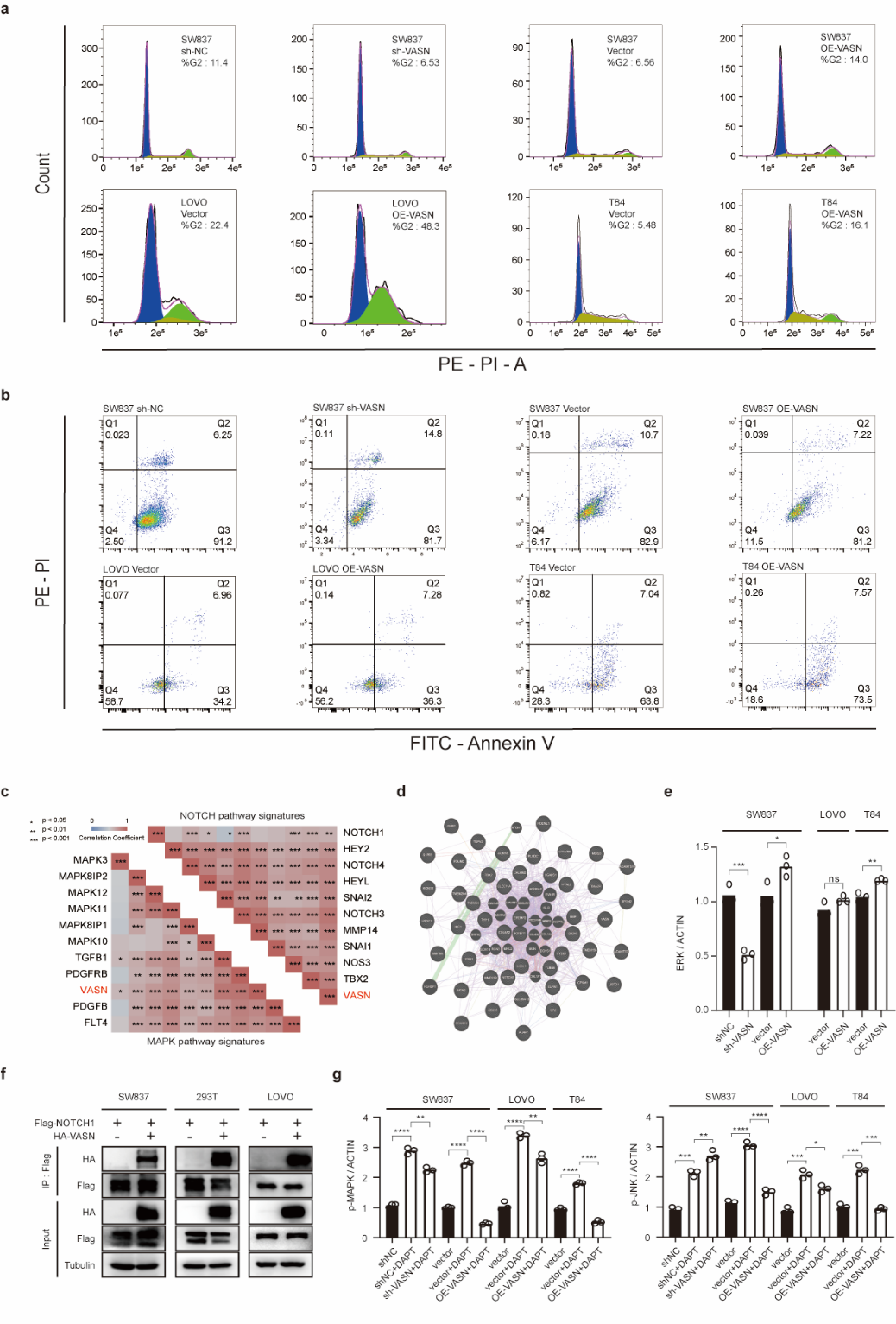


**a**, Flow cytometry experiments showed that VASN overexpression increased and knockdown decreased the proportion of G2 phase cells in CRC cell lines. **b,** Annexin V/PI staining detected cell apoptosis in CRC cell lines treated with 5-FU. **c,** Correlation heatmap of the top 10 genes with the highest correlation to VASN in MAPK and NOTCH pathways. **d,** Proteins co-expression network from GeneMANIA dataset illustrated the correlation of VASN with MAPK and NOTCH pathway-related proteins. **e,** Quantitative result of ERK protein expression in western blot analysis of VASN-intervened cells. **f,** The exogenous co-immunoprecipitation (co-IP) experiment confirmed the binding of VASN with the NOTCH1 protein. **g,** Quantitative Western blot analysis of phosphorylated proteins in VASN-intervened cells under DAPT treatment. *, P value < 0.05; **, P value < 0.01; ***, P value < 0.001.
