# Supplementary material for: Vasorin (VASN) overexpression promotes pulmonary metastasis and resistance to adjuvant chemotherapy in patients with locally advanced rectal cancer
Source: J Transl Med. 2024 Aug 6;22:742. doi: 10.1186/s12967-024-05473-4 (PMC11301854; doi:10.1186/s12967-024-05473-4)
Supplement: Supplementary file 2 — Supplementary Material 2 [file 12967_2024_5473_MOESM2_ESM.docx]

Dear Editors of *Journal of Translational Medicine*,

We appreciate your thorough review, valuable feedback, and constructive suggestions, all of which have greatly enhanced the quality of our manuscript presentation. We have thoroughly reviewed the feedback provided by the reviewers and made appropriate revisions to our manuscript. Additionally, we have conducted a meticulous proofreading process to rectify any typos and grammatical errors we identified. We have studied these comments carefully and have made corrections which we hope to meet with approval. The changes are marked in red in the revised manuscript. The main corrections in the paper and the responses to the reviewer’s comments are as follows.

***Reviewer #1:***

*1. Figure 2 Authors observed that knocking down VASN significantly suppressed the proliferation, migration, and invasion of colorectal cancer (CRC) cells, while overexpression of VASN exerted an opposing effect. It would be beneficial if the authors could further investigate the expression of protein markers related to proliferation and migration to provide* *additional insights into the biological mechanisms involved.*

**Response:** Thank you for your suggestion. In this revised version, we have included the Western blot experiment results for metastasis-related proteins (N-cadherin, E-cadherin, β-catenin) and proliferation-related proteins (CDK1, cyclin B1). We found that VASN expression is positively correlated with the expression of N-cadherin and CDK1, which explains the influence of VASN on cell proliferation and metastasis (Supplemental Figure 1j).

*2. Some studies have shown that VASN is associated with anti-apoptosis. Have the authors investigated the effect of VASN on cell cycle distribution and apoptosis?*

**Response:** Thank you for your suggestion. Following your advice, we have included the flow cytometry experiments in this revised version (Supplemental Figure 2a-2b). The results showed that overexpression of VASN can drive CRC cells into the G2 phase, which is consistent with the WB results for CDK1, indicating that VASN can regulate the cell cycle to promote cell proliferation. In the apoptosis experiment, cells were initially treated with 5-FU for 72 hours and the subsequent staining results revealed that overexpression of VASN induced less proportion of apoptotic cells and decreased drug sensitivity in rectal cancer cell line SW837. However, significant changes were not observed in the colon cancer cell lines LOVO and T84, possibly due to tumor heterogeneity.

*3. Authors concluded that VASN was significantly increased in CRC cell lines compared with cell line NCM460, Nevertheless, the knockdown of VASN was performed using just a single CRC cell. Additionally, there was inconsistency in the cell lines utilized in Figure 2-5.*

**Response:** Thank you for your suggestion and we have added the relevant experiments with LOVO and T84 cells as requested (Supplementary Figure 1e-1i). We also have provided some clarification on the previous cell line selection in the initial version: This study mainly focuses on the expression of VASN on the biological functions in LARC patients with lung metastasis. Therefore, we generated stable rectal cancer cell line, SW837, with overexpression and knockdown of VASN to explore potential molecular mechanisms. In parallel, the LOVO and T84 colon cancer cell lines were used to compare the heterogeneity between colorectal cancer. Given the impact of low VASN baseline expression levels (Supplementary Figure 1C) on the efficiency of knockdown cell line construction in LOVO and T84 cells, we initially established VASN overexpression cell lines for these cell lines.

After addressing the issue above, the inconsistency in cell lines you mentioned mainly revolves around the use of the 293T cell line in the IP assays (Figure 4f, 4h). The choice of 293T cell line was based on its ease of transfection, facilitating the observation of the widespread nature of protein interactions across different cell types. Similar approaches have been widely applied in protein-related studies, and we have provided a brief explanation in the "co-IP assays " section of Methods.

*4. VASN is an oncogene. How does the author interpret the significantly higher proportion of patients with VASN-negative (N=68) compared to those with VASN-positive (N=30)?*

**Response:** Thank you for your question and it is a good point. It is generally acknowledged that tumor metastasis is a multi-step process, where molecular changes are dynamic and continuous. In our study, VASN expression increased with advancing clinical stages (Figure 1), particularly in stage IV patients with lung metastasis. Our study indicated that VASN-positive patients are more prone to experiencing lung metastasis events, resulting in a higher mortality rate in these patients. It suggested that VASN may play a more significant role in advanced metastatic tumors, rather than early stages. In our IHC scoring analysis, we also quantified regional expression using the HALO software. During this process, we identified and excluded vascular tissues with high VASN expression, focusing solely on tumor epithelial cells. Furthermore, considering the false positive results due to non-specific staining during the IHC process, we applied strict thresholds for region quantification. During this process, vascular VASN expression and weaker staining signals were excluded, which could be one of the reasons for the differences in group proportions. Our results showed that most of the patients with primary tumors exhibited negative VASN expression, while the pulmonary metastasis rate in the VASN-positive group reached 90% (Table 1). Although the random selection of fields of view and tumor samples might introduce bias, it is more importantly highlighted that VASN plays a pro-metastatic role in late stage rather than early LARC patients.

*5. Authors found VASN regulates the MAPK pathway through the NOTCH1 protein. How do the MAPK pathway and the NOTCH1 protein contribute to pulmonary metastasis and resistance to adjuvant chemotherapy in patients with locally advanced rectal cancer?*

**Response:** Thank you for your question. The MAPK and NOTCH pathways have complex regulatory networks in biological processes, both playing significant roles in the development and progression of tumors. In the rescue experiment, we treated stable cell lines of different VASN states with the NOTCH pathway inhibitor DAPT. The results indicated that VASN partially rescues the inhibitory phenotype induced by DAPT, but overall remains suppressed compared to the control group. Meanwhile, immunofluorescence (IF) experiments have confirmed a direct interaction between VASN and NOTCH1 proteins, and Western blot (WB) assays have shown that they can upregulate proteins expression mutually. Additionally, previous studies have reported an interaction between VASN and NOTCH1 proteins in glioma, contributing to the maintenance of tumor cell stemness. Considering the background mentioned above, we believe that the NOTCH pathway plays a more significant role in promoting the metastasis and drug resistance (stemness) of rectal cancer, while the MAPK pathway is involved in cell growth and proliferation.

*6. The author could preferably provide a mechanistic diagram for this study to facilitate readers' quick understanding.*

**Response:** Thank you very much for your suggestion. To help readers grasp the main idea of this article quickly, we have added a brief mechanistic diagram at the end of this study to illustrate the oncogenic effect of VASN (Figure 7).

*7. Regarding statistical analysis, it is crucial to conduct normality and frequency tests to validate the appropriateness of using the Student's t-test and ANOVA.*

**Response:** Thank you very much for your statistical suggestions. All the statistical analyses in this study were performed after assessing normality and frequency tests of the data. The P value was calculated using t-test or Wilcoxon rank sum test for two groups and ANOVA for multiple groups. These details have been supplemented in the " Statistical analysis" section of Methods.

*8. The number of replicates used for the analysis has not been specified. Additionally, the use of bar graphs is discouraged due to their limitation in providing meaningful insights.*

**Response:** Thank you for your comments. All our experiments were conducted with three or more replicates, and statistical tests were performed using quantified values, as clarified in the "Statistical Analysis" section. Regarding the modification of the bar graphs, this is a very good suggestion. For comparisons between groups with a large sample size (N > 5), we have re-visualized using violin plots, which allows readers to easily observe the distribution of the data (Figure 1, Figure 6). At the same time, considering the limited number of replicates in the experimental results (typically three times), we have added floating dots on the bar plot to indicate specific values (Figure 2-5).

*9. Figures 2-5 are missing significance analysis for some data points, and the charts also lack annotations indicating sample sizes.*

**Response:** Thank you so much for your careful check. We have supplemented some missing significance analysis and labeled the sample sizes from different groups, allowing readers to observe the comparisons scale directly.

*10. The* *microscopic images do not include scale bars, which are essential for accurate interpretation.*

**Response:** We are very sorry for our negligence of checking the image versions. We have observed some microscopic images are missing a scale bar, and we have addressed this issue in the latest revision.

*11. Figure 2g is unclear. Could you please provide a more legible image?*

**Response:** Thank you for your comments. Considering the size limitations of the uploaded files, we have placed the images below for display.

**Reviewer #2:**

*1. Since the topic is related to early or locally advanced rectal cancer patients, it would be important to better describe and define baseline patient's and tumour characteristics in terms of clinical stage at diagnosis according to TNM classification (i.e. I-II-III) and MRI radiological risk factors (i.e. mesorectal fascia (MRF) involvement, lateral pelvic lymph node (LPLN) status, extramural vascular invasion (EMVI) presence, circumferential resection margin (CRM) status), if available. Indeed, it woul be interesting to understand if certain well defined radiological* *characteristics may predict a VASN positivity.*

**Response:** Thank you very much for your comments. Investigating the association of VASN expression with clinical characteristics in LARC patients is a valuable suggestion. We also noted this during the data collection phase, but due to the extensive treatment timelines (nearly 10 years) and variations in treatment approaches, the treatment processes for most metastatic patients were quite complex. Consequently, a significant portion of the imaging-related information for these patients was missing, which is regrettable. Nevertheless, we made efforts to collect the information in this revised version. Considering the clinical significance of your suggestion, we intend to undertake relevant retrospective work in future. Once again, thank you for your suggestion.

*2.* *At pag 14 the following sentence: "Analysis of the collected data revealed that middle and low rectal cancer patients had a greater incidence of pulmonary metastasis (lung 6.3% vs. liver 4.6%) (Figure 1a) and a worse five-year overall survival (OS) rate than patients without pulmonary metastasis (metastasis 37.1% vs. primary 89.8%)" has no sense. Compared to whom does patients with middle and low rectal cancer have a greater incidence of lung mets? (maybe compared to pts with colon cancer? Or compared to pts with upper rectal cancer?). Moreover, it is obvious that pts with lung mets have worst OS compared to pts without lung mets (i.e. with primary tumour only), isn't it? I suggest the authors to rephrase the sentence.*

**Response:** Thank you for your suggestion. Previous studies have shown that patients with rectal cancer are more prone to developing lung and neural metastases compared to those with colon cancer; notably, patients with mid-low rectal cancer have the highest incidence of lung metastasis among all CRC patients (Reference 14,15). Furthermore, LARC patients with lung metastases generally have a worse clinical prognosis, a common challenge for metastatic patients. This section aims to emphasize the preference for lung metastases in rectal cancer and its adverse prognosis. Following your advice, we have rephrased this sentence to improve clarity for the readers.

*3.* *It is not clear if high levels of VASN correlate with a higher probability of developing lung metastasis in a synchronous or in a metachronous way with respect of the primary tumour diagnosis. Moreover, it is not clear how the patient's selection has been performed according to each analysis. To this instance, I suggest the author to better describe the population of rectal cancer patients involved in the study with the use of a CONSORT flow diagram.*

**Response:** Thank you for your suggestion. We have added the metastatic data (primary, synchronous and metachronous) in Table 1. Additionally, we have created a simple flow diagram to describe the population of rectal cancer patients involved in the study: We accessed data from patients diagnosed with LARC at SYSUCC dataset over the past 10 years (2008-2018). Subsequently, we divided the patients into two groups based on their lung metastasis status and used TNM staging for propensity score matching of the two groups. Finally, we selected samples from the SYSUCC pathology resource repository for IHC scoring.

*4. Besides the description of the OS results, it would be important to define the median follow-up time and the number of OS events at that time. Of course, the number of events as described in table 1 seems to be quite heterogeneous (24% vs 53% in the VASN-negative and VASN-positive cohort, respectively).*

**Response:** Thank you for your suggestion and we have added the follow-up time information in Table 1.

*5. The table 2 (uni- and multi-variate analysis for OS) should be better outlined and explained since the (at least) dichotomus categorization of each variable is not reported. Thereafter it is not possible to understand the prognostic role (if positive or negative) of each variable assessed.*

**Response:** Thank you for your suggestion. We have re-described the variables in both the univariate and multivariate cox analysis, and we have labeled the HR values for each variable in Table 2.

*6. In rectal cancer, particularly in medium and lower locally advanced rectal cancer, the standard of care management according to ESMO and NCCN guidelines should include a chemoradiation treatment together with systemic chemotherapy before (as induction treatment) or after (as consolidation treatment) the chemoradiotherapy. The rationale of the so-called "total neoadjuvant therapy" (TNT strategy) is related to the absence of evidence of a survival benefit (in terms of OS) by adjuvant chemotherapy in patients affected by rectal cancer, and its aim would be to limit the risk of metastatic disease, to increase the probability of reaching a pathological complete response (pCR) which is in turn related to a better prognosis and to the possibility to avoid conservative surgery (i.e. rectal anterior resection) in pts achieving a complete clinical response (cCR) at the MRI radiological reassessment after TNT. In the present study the great majority (at least the 2/3) of the patients with rectal cancer, despite defined as "LARC", were treated with upfront surgery, with no radiotherapy, chemotherapy or chemoradiation therapy. I suggest the authors to disclose and discuss this as limitation in the discussion section.*

**Response:** Thank you for your suggestion. The patients in our cohort had a wide range of diagnosis and treatment timelines, with variations in the treatment approaches for patients across different periods. Additionally, the treatment process for metastatic patients is more complex. Therefore, much of the treatment information is missing. Following your advice, we have reinserted a discussion of the data limitations in the revised version.
